# Inhibition of p38 MAPK in combination with ART reduces SIV-induced immune activation and provides additional protection from immune system deterioration

**DOI:** 10.1371/journal.ppat.1007268

**Published:** 2018-08-30

**Authors:** Omkar Chaudhary, Vivek Narayan, Felipe Lelis, Brandon Linz, Meagan Watkins, Ronald Veazey, Anna Aldovini

**Affiliations:** 1 Boston Children’s Hospital, Department of Medicine, and Harvard Medical School, Department of Pediatrics, Boston MA, United States of America; 2 Tulane National Primate Research Center, Division of Comparative Pathology, Covington LA, United States of America; University of Illinois at Chicago College of Medicine, UNITED STATES

## Abstract

Differences in immune activation were identified as the most significant difference between AIDS-susceptible and resistant species. p38 MAPK, activated in HIV infection, is key to induction of interferon-stimulated genes and cytokine-mediated inflammation and is associated with some of the pathology produced by HIV or SIV infection in AIDS-susceptible primates. As small molecule p38 MAPK inhibitors are being tested in human trials for inflammatory diseases, we evaluated the effects of treating SIV-infected macaques with the p38 MAPK inhibitor PH-797804 in conjunction with ART. PH-797804 had no side effects, did not impact negatively the antiviral immune response and, used alone, had no significant effect on levels of immune activation and did not reduced the viremia. When administered with ART, it significantly reduced numerous immune activation markers compared to ART alone. CD38^+^/HLA-DR^+^ and Ki-67^+^ T-cell percentages in blood, lymph node and rectal CD4^+^ and CD8^+^ T cells, PD-1 expression in CD8^+^ T cells and plasma levels of IFNα, IFNγ, TNFα, IL-6, IP-10, sCD163 and C-reactive protein were all significantly reduced. Significant preservation of CD4^+^, CD4^+^ central memory, CD4^+^/IL-22^+^ and CD4^+^/IL-17^+^ T-cell percentages and improvement of Th17/Treg ratio in blood and rectal mucosa were also observed. Importantly, the addition of PH-797804 to ART initiated during chronic SIV infection reduced immune activation and restored immune system parameters to the levels observed when ART was initiated on week 1 after infection. After ART interruption, viremia rebounded in a similar fashion in all groups, regardless of when ART was initiated. We concluded that the inhibitor PH-797804 significantly reduced, even if did not normalized, the immune activation parameters evaluated during ART treatment, improved preservation of critical populations of the immune system targeted by SIV, and increased the efficacy of ART treatment initiated in chronic infection to levels similar to those observed when initiated in acute infection but did not affect positively or negatively viral reservoirs.

## Author summary

The hallmark of Human Immunodeficiency Virus and Simian Immunodeficiency Virus infection in disease-susceptible species is the progressive decline of the CD4+ T cell population and heightened immune activation, which by itself can contribute to CD4+ T-cell death. The cellular pathway regulated by p38 MAPK, which is activated in HIV and SIV infection, can contribute significantly to immune activation. We tested in SIV-infected macaques a p38 MAPK inhibitor in combination with anti-retroviral therapy. This drug is already being evaluated in humans for treatment of immune activation associated with other diseases. We found that, when combined with antiretroviral therapy, the inhibitor PH-797804 significantly reduced a few parameters of SIV-induced immune activation and improved preservation of critical populations of the immune system targeted by SIV, but did not modulate viral reservoirs. Importantly, the addition of the inhibitor to anti-retroviral therapy during the chronic phase of the infection, which is the time when most HIV-infected individuals initiate treatment, permitted a more significant preservation of the immune system compared to antiretroviral therapy alone that was similar to that observed when anti-retroviral therapy was initiated in the acute phase of the infection, which rarely occurs in HIV infection.

## Introduction

Differences in immune activation have been identified as the single most significant difference between AIDS-susceptible and resistant species [1–9]. Immune activation can be induced by a variety of mechanisms, including stimulation of innate and adaptive immune responses, production of a superantigen, and/or production of activating cytokines and chemokines. It is quite likely that more than one mechanism is occurring simultaneously during HIV infection. As immune activation can trigger T-cell apoptosis, the differential level of immune activation induced by HIV and SIV among the species could explain the more drastic depletion of CD4^+^ T cells that occurs in AIDS susceptible compared to AIDS-resistant species, as apoptosis occurs significantly less in species that do not develop AIDS [7, 10, 11]. Apoptosis and immune activation are substantially reduced in HIV-infected individuals that are long-term non-progressors [12, 13]. Residual chronic immune activation during ART is considered a contributor to the co-morbidity observed during treatment, mainly accelerated aging-related diseases, including renal dysfunction, atherosclerosis and hypertension, diabetes mellitus, respiratory diseases (e.g. chronic obstructive pulmonary diseases and pneumonia), and HIV-associated neurological disorders [14–17].

Inhibition of immune activation has been explored in a few studies that have targeted different molecules and pathways. A COX-2 inhibitor tested in 13 HIV-infected individuals appeared to reduce immune activation as indicated by reduction of PD-1 on CD8^+^ T cells, increasing numbers of T regulatory (Treg) cells, and improved recall responses to a T-cell dependent vaccine [18]. p38 MAPK is also involved in the activation of COX-2 and inhibition of p38 MAPK also resulted in the inhibition of COX-2 [19]. However, when tested in a randomized placebo-controlled trial, no significant immunological effects of the COX-2 inhibitor Etoricoxib were observed in ART-treated patients [20]. PD-1 blockade also reduced immune activation [21]. Interestingly, it is p38 MAPK that stimulates the transcription of PD-1 and induces additional molecules inhibitory of T-cell function [22, 23]. Anti-TNFα treatment during SIV infection, whose expression is dependent on p38 MAPK, also reduced immune activation [24]. Instead, blocking IFNα in pDC did not reduce immune activation in SIV infected macaques and the administration of an IFNα agonist in SIV-infected sooty mangabeys did not result in immune activation, making it somehow less likely that the direct activity of IFNα is the cause of immune activation in Rhesus macaques (RM) [25, 26]. Reduced inflammation was observed in SIV-infected RM when ART was combined with IL-21, which also impacted time to rebound, plasma viremia and cell-associated SIV DNA levels after ART interruption but the mechanism of this outcome was not fully understood [27].

Induction of interferon-stimulated genes (ISG) during a viral infection is a consequence of Toll-like receptor (TLR) activation [28]. This leads to increased transcription of IRF7 and triggering of IFN**α** production, activation of the kinase cascade, with up-regulation, among others, of p38 MAPK. p38 MAPK triggers a signaling pathway that leads to direct activation of transcription factors implicated in many of cellular process including inflammation, cell cycle, apoptosis, and immune response [29, 30]. The p38 MAPK pathway is critical for maintaining a sustained response to type I and type II IFNs, leading to the induction of transcription of ISGs via activation of signal transducer and activator of transcription (STAT) proteins [31–35]. In addition, p38 MAPK plays an important role in regulating IFN-independent transcription of some ISG after TLR7 triggering [36–39] and production of inflammatory cytokines such as IL-1 and tumor necrosis factor alpha (TNF**α**). Inhibition of the p38 MAPK pathway may form the basis of a new strategy for treatment of inflammatory diseases.

p38 MAPK plays crucial roles in various pathological processes associated with HIV infection, including macrophage activation, neurotoxicity and impairment of neurogenesis, and lymphocyte apoptosis [29, 30]. Increased, active p38 MAPK has been reported in brains of SIV-infected macaques with encephalitis [40]. Interestingly, p38 MAPK has also been implicated in the production and release of IP-10 in astrocytes exposed to HIV-1 and Tat [41–43]. HIV-1 and Tat were reported to activate p38 MAPK in infected or stimulated monocytes and macrophages [44]. We have shown that HIV and SIV Tat modulates primate antigen presenting cells (APC) and that at least a subset of the ISG are not equally affected by SIV infection in APC of AIDS resistant species [45–47]. We found that Tat associated with the MAP2K6, MAP2K3 and IRF7 promoters and that the association resulted increased activation of p38 MAPK and consequent induction of ISG [45–47]. This mechanism of p38 MAPK activation could further and independently chronically contribute to the activation of ISG that results from TLR activation. Collectively, these data indicate that p38 MAPK activation is an important, additional mediator of HIV-associated pathology. Evaluating *in vivo* the role played by p38 MAPK in HIV replication and immune activation by inhibiting its activity may provide the rationale for the use of p38 MAPK inhibitors in AIDS therapy, in association with ART or when ART is no longer an option.

A series of compounds targeting p38 MAPK were initially discovered and were followed by the development of more potent and specific inhibitors of this protein capable of inhibiting the production of inflammatory cytokines. Different p38 MAPK inhibitors have been shown efficacy in preclinical animal models of a variety of diseases [48–56]. Some of these inhibitors have advanced to clinical studies for rheumatoid arthritis, chronic obstructive pulmonary disease (COPD), post-herpetic neuralgia and neuropathic pain, and osteoarthritis. [54, 57–59]

The diarylpyridinone PH-797804 is a novel, ATP-competitive and reversible potent inhibitor of human p38 MAPK [53]. It specifically inhibits p38α with IC_50_ value of 26 nM and K(i) value of 5.8 nM and inhibits LPS induced TNFα and IL-1β production in monocytes in a concentration-dependent manner [50]. PH-797804 blocks RANKL and M-CSF induced osteoclast formation in primary rat bone marrow cells. When given orally, PH-797804 reduces TNFα levels in LPS-induced shock of Lewis rats and of cynomolgus monkeys [50]. In randomized, adaptive design, double-blind, placebo-controlled, parallel-group, multicenter trial demonstrated improvements over placebo in lung function parameters and dyspnea in patients with moderate to severe COPD [58, 60].

Here we show that inhibiting p38 MAPK *in vivo* can significantly impact SIV-mediated immune activation and protect immune cell populations that are negatively affected by the infection. However, the reduction of immune activation is not complete, it is unlikely to be fully controlled by acting on any single activation pathway, and possibly requires exploration of different inhibitor doses and intervention on multiple pathways linked to immune activation, including other kinases like JNK that are also affected by HIV and SIV.

## Results

### Effectiveness of ART and p38 MAPK inhibitor treatment

**Fig 1:**
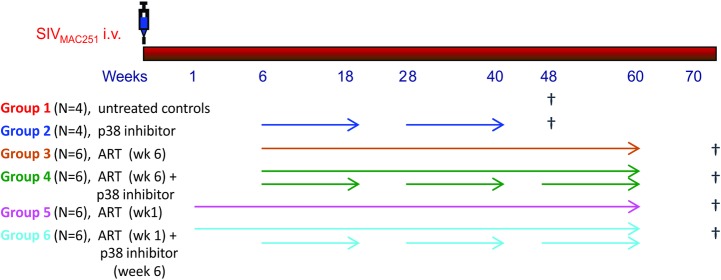
Experimental design. Group treatments are illustrated and color-coded, with the color used for the group also used in the subsequent figures to identify the data for each individual group. Animals in Groups 1 and 2 were euthanized on week 48, as no significant differences of viremia and immune activation were observed between the two groups during the time course. All treated animals were monitored for 20 weeks after ART interruption.

The contribution of p38 MAPK in chronic immune activation during lentiviral infection was investigated in the SIV-infected RM animal model, treated with ART over the course of 60 weeks. RM were infected intravenously (i.v.) with 10 TCID50 SIVmac_251_ and divided in groups (n = 4, Groups 1 and 2; n = 6, groups 3–6), with some groups receiving ART alone and others ART combined with p38 MAPK inhibitor (Fig 1). ART consisted of two reverse transcriptase (RT) inhibitors, tenofovir (PMPA, 20 mg/kg, and emtricitabine (FTC, 30 mg/kg), and the integrase inhibitor dolutegravir (DTG, 2.5mg/kg) s.i.d, administered i.m. and was initiated in the acute phase of the infection, one week after SIV infection, in groups 5 and 6, or in the chronic phase, once set point was reached, six weeks post-infection, in Groups 3 and 4. PH-797804, 10 mg s.i.d, orally administered, was chosen among other similar compounds for the proposed studies for multiple reasons. It has been tested in humans and cynomolgus monkeys where it could effectively inhibit the acute inflammatory response that follows the administration of lipopolysaccharide (LPS), in particular production of TNFα and IL-6, and it reduced chronic inflammation and bone loss associated with arthritis in mice and rats [50]. In cynomolgus monkeys exposed to a single dose of LPS, PH-797804 dosed intragastrically at 0.001 mg/kg to 1 mg/kg, the dose of 0.1 mg/kg reduced TNFα plasma levels to 20% of the levels observed in animals treated with placebo, while the dose of 1 mg/kg reduced it to less than 10% [50]. This reduction was virtually identical to that observed for TNFα and IL-1β in humans after LPS challenge, where a reduction of 50% of the IL-6 levels was also observed [50]. As primary endpoints, we evaluated differences in expression of surface and intracellular molecules linked to immune activation and plasma levels of inflammatory cytokines. As secondary endpoints, we evaluated the effects that treatments had on viral loads, preservation of central memory (C_M_) and other CD4^+^ T cell subpopulations.

**Fig 2:**
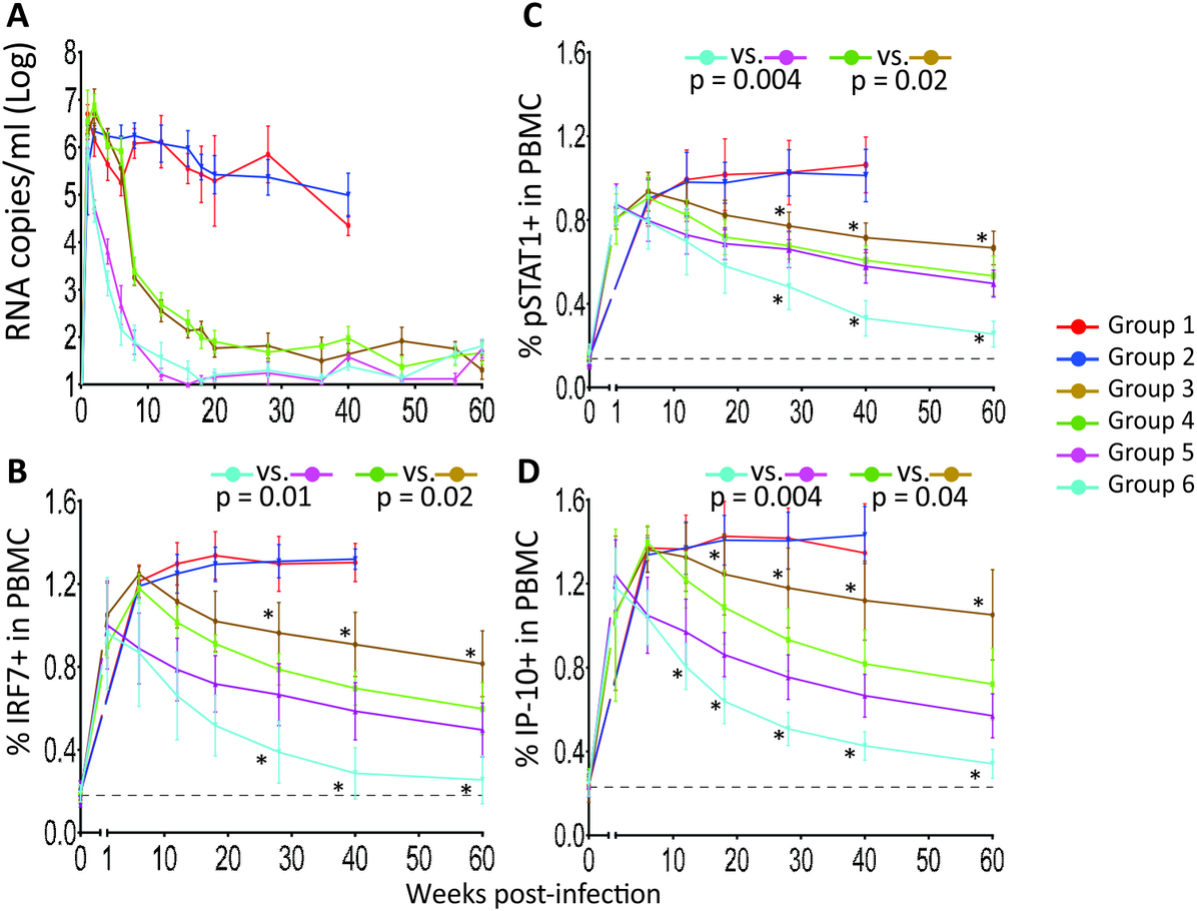
Evaluation of treatment efficacy. A. Plasma viral loads are reported as RNA copies/ml and are shown group average (mean ± SEM). Longitudinal assessment of intracellular expression of three ISGs in PBMC by flow cytometry: B. IRF7, C. pSTAT1, D. IP-10. Data are reported as percentage of positive PBMC and are shown as group average (mean ± SEM) (solid line). The reported p values were calculated for the comparison of the AUC from week 8 to 60 and refer to AUC comparisons in paired groups. Between groups comparisons at individual time points were carried out with Wilcoxon-Mann-Whitney (rank sum) test. Asterisks mark significant time point paired comparisons for Group 3 vs. Group 4 (asterisks above brown line) or Group 5 vs. Group 6 (asterisks below blue line). The black, dotted line indicates the average of all 32 individual animal values measured before infection.

After SIV_mac251_ infection all animals experienced a rapid increase in viremia that peaked before initiation of ART for the groups when ART was initiated on week 6 and was below peak levels in the groups initiating ART one week after infection. ART was effective in suppressing the viral replication in all animals that received it (Fig 2A). The p38 inhibitor PH-797804 was well tolerated and no major side effects were noticed throughout the study. At the dose used in these animals (10 mg s.i.d, orally), plasma viral loads were comparable between groups receiving PH-797804 and those that did not, indicating that the p38 MAPK inhibitor did not affect virus replication, whether administered alone or in combination with ART, and regardless of when ART was initiated. To obtain a preliminary indication of PH-797804 efficacy *in vivo*, and considering that p38 MAPK does not directly affect ISG expression but does so via directly increasing the activity of the master transcription regulators of ISG expression IRF7 and pSTAT1, we evaluated the percentages of PBMC positive for accumulation of IRF7 and pSTAT1, and the cytokine IP-10, one of the ISG most significantly upregulated in HIV and SIV infections (Fig 2B–2D, S1A Fig). Using intracellular staining and flow cytometry, we found that the accumulation of these proteins was significantly reduced by the inhibitor when percentages from animals receiving ART alone were compared to those receiving ART and inhibitor for the two paired groups, whether initiating ART at week 1 or 6 (area under the curve from week 8 to 60, IRF7: p = 0.01, pSTAT1: p = 0.004, IP-10: p = 0.004 for groups initiating ART at week 1 and IRF7: p = 0.02, pSTAT1: p = 0.02, IP-10: p = 0.04, for groups initiating ART at week 6). This was true whether the analysis was done on total PBMC or CD3^+^ T-cell subpopulations (S1C Fig). Percentages of the CD3^+^ subpopulations were comparable at individual time points in paired groups (S1B Fig). Results were very similar when the same analyses were carried out in lymph node mononuclear cells (MNC) (S1D Fig). This result indicates that the selected PH-797804 dose could reduce expression of ISG transcriptional regulators and of the cytokine IP-10.

**Fig 3:**
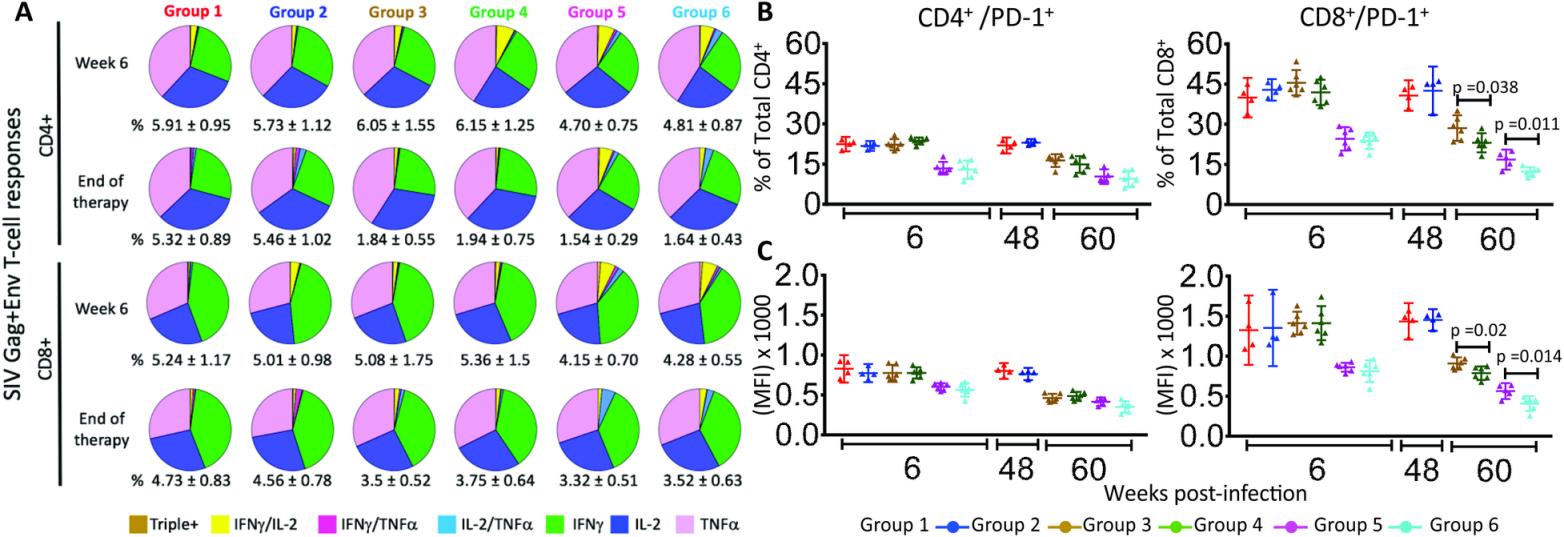
SIV-specific cell-mediated immune responses and PD-1 expression in PBMC T cells. A. Analyses for week 6 and week 60 after infection are shown. Group mean percentages of SIV-(Gag+Env)-specific CD4^+^ or CD8^+^ T cells of total CD4^+^ or CD8^+^ T cells ± SEM are indicated below each pie graph. The functional diversity of the SIV-specific responses is further illustrated with pie graphs for each group. Individual colors within each pie graph are sized according to the size of each fractions of the total SIV-specific CD4^+^ and CD8^+^ T-cells (# reported below the pie graph) producing one or simultaneously two or three cytokines after peptide stimulation. IFNγ^+^/IL-2^+^/ TNFα^+^, brown; IFNγ^+^/IL-2^+^/ TNFα^−^, yellow; IFNγ^+^/IL-2^-^/ TNFα^+^, dark pink; IFNγ^+^/IL-2^-^/ TNFα^-^, green; IFNγ^−^/IL-2^+^/ TNFα^+^, light blue; IFNγ^−^/IL-2^+^/ TNFα^−^, dark blue; IFNγ^−^/IL-2^−^/ TNFα^+^, light pink. B. and C. Percentages and Median Fluorescence Intensity (MFI) of PD-1^+^/CD4^+^ and PD-1^+^/CD8^+^ T cells in PBMC CD4^+^ and CD8^+^ T cells at week 6 and 48 (Groups 1 and 2) or 60 (Groups 3–6) post-infection are shown as scatter plots and 95% confidence interval. Unpaired *t* test was used for the comparison between two groups.

To exclude that the inhibitor treatment could reduce the effectiveness of the antiviral immune response, we evaluated the levels of antigen-specific cell mediated responses over the course of the treatment. We found that the number of SIV-specific CD4^+^ and CD8^+^ T cells were similar in the group pairs and proportionate to the viral loads present in the animals (Fig 3A), indicating that the p38 inhibitor treatment did not grossly altered the magnitude of the anti-viral immune response. We also evaluated whether inhibition of p38 MAPK could impact the expression of PD-1, checkpoint known to increase during infection because of persistent immune activation, resulting in inefficient CD8^+^ T-cell activity [61]. When investigated on week 60, before removal of ART and inhibitor treatment, we found that the frequency of CD8^+^ T cells expressing PD-1 was significantly lower in the groups that received the p38 inhibitor combined with ART compared to ART alone, whether ART treatment started on week 1 (p = 0.011) or 6 (p = 0.038) whereas it was comparable in CD4^+^ T cells (Fig 3B and 3C). We concluded that PH-797804 did not affect negatively the development of anti-SIV immune responses and reduced the expression of the checkpoint inhibitor PD-1 in CD8^+^ T cells, most likely indirectly via the overall impact on immune activation.

### Impact of PH-797804 on immune activation in PBMC and tissues

**Fig 4:**
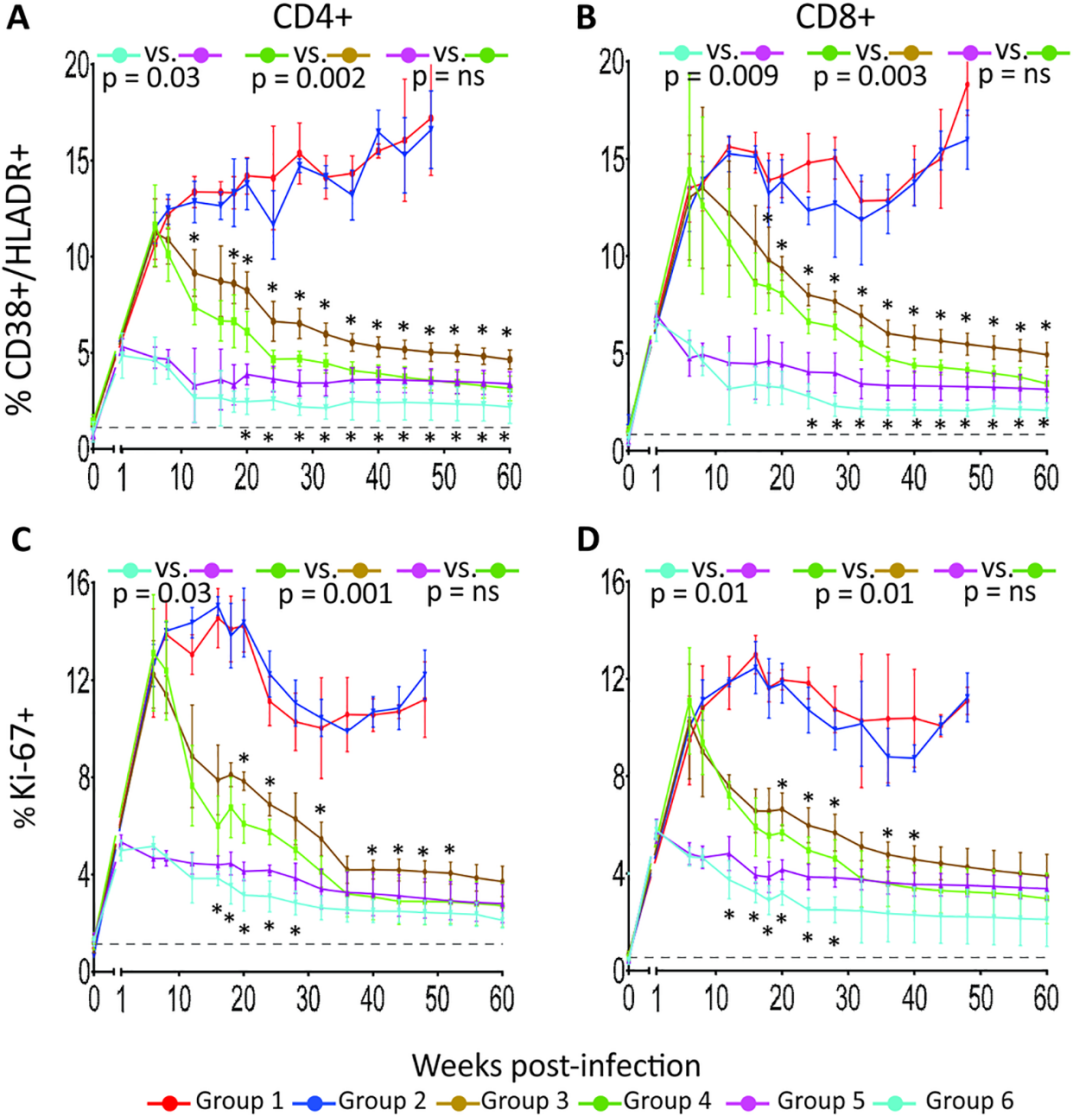
Longitudinal analysis of immune activation marker expression in PBMC T cells of SIV-infected and treated or untreated RMs. Percentages of HLADR^+^/CD38^+^ in CD4^+^ (A) and CD8^+^ (B) T cells and of Ki-67^+^ in CD4^+^ (C) and CD8^+^ (D) T cells in PBMC. Data are reported as group average (mean ± SEM) (solid line). The black, dotted line indicates the average of all 32 individual animal values measured before infection. The reported p values were calculated for the comparisons of the AUC from week 8 (first available time point after beginning of PH-797804 treatment) to 60 and refer to AUC comparisons in paired groups. Between groups comparisons at individual time points were carried out with Wilcoxon-Mann-Whitney (rank sum) test. Asterisks mark significant time point paired comparisons for Group 3 vs. Group 4 (asterisks above brown line) or Group 5 vs. Group 6 (asterisks below blue line).

**Fig 5:**
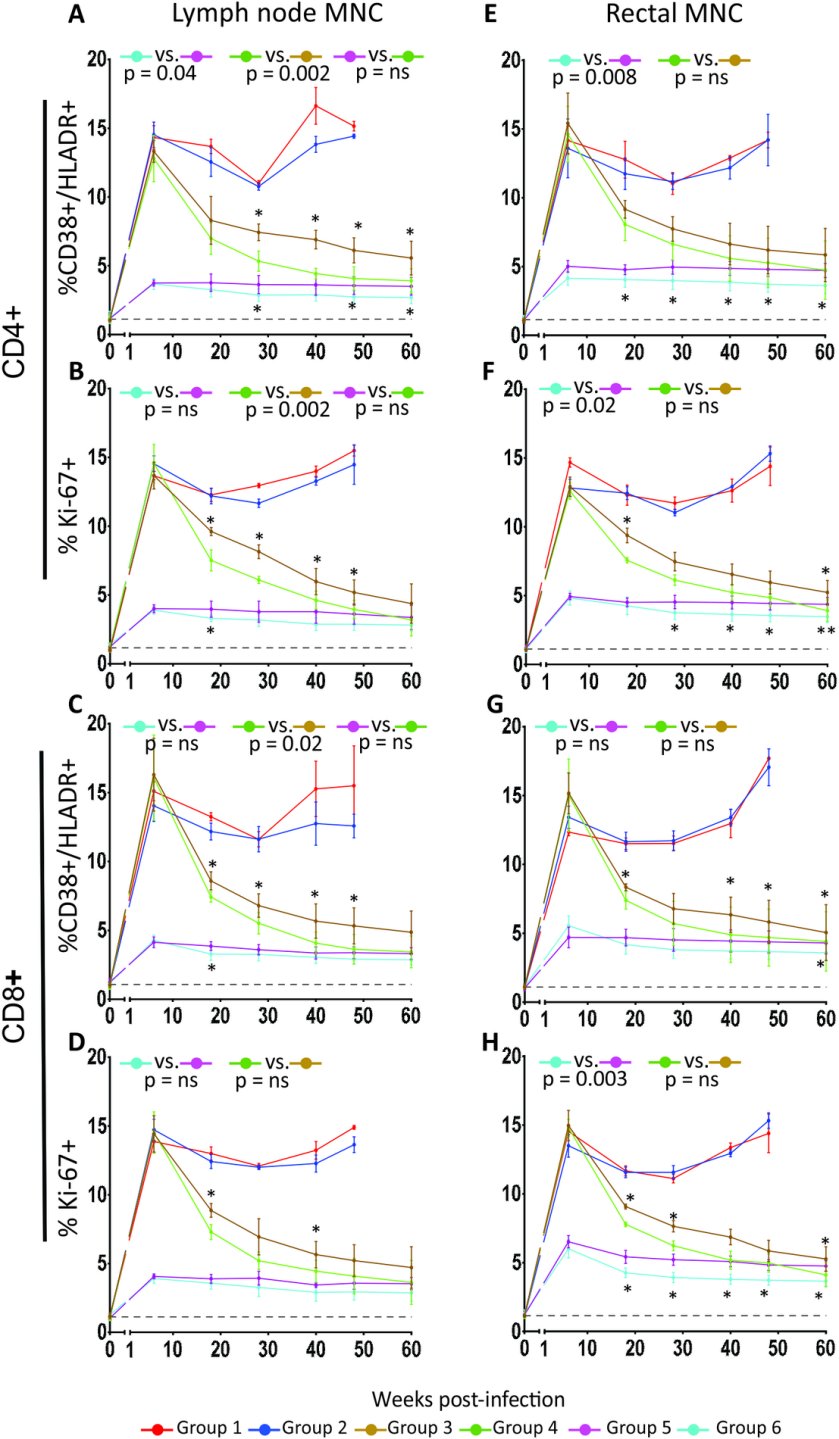
Longitudinal analysis of immune activation marker expression in tissue T cells of SIV-infected and treated or untreated RMs. Data for lymph node and rectal tissue T-cell expression of immune activation markers, in biopsies collected at each PH-797804 treatment cycle start and end time points are shown. Panels report percentages HLADR^+^/CD38^+^/CD4+ (A) or Ki-67^+^/CD4^+^ T cells (B) in inguinal lymph nodes and in rectal mucosa (E and F, respectively), percentage of HLA-DR^+^/CD38^+^/CD8^+^ (C) or Ki-67^+^/CD8^+^ T cells (D) in lymph nodes and in rectal mucosa (G and H, respectively). Data are represented as group mean ± SEM. The black, dotted line indicates the average of all 32 individual animal values measured before infection. The reported p values were calculated for the comparison of the AUC from week 18 (first available time point after beginning of PH-797804 treatment) to 60 and refer to AUC comparisons in paired groups. Between groups comparisons at individual time points were carried out with Wilcoxon-Mann-Whitney (rank sum) test. Asterisks mark significant time point paired comparisons for Group 3 vs. Group 4 (asterisks above brown line) or Group 5 vs. Group 6 (asterisks below blue line).

Chronic immune activation has been proposed to be a key determinant of AIDS pathogenesis. A variety of cell surface determinants expressed in the cell membrane are phenotypically associated with T-cell immune activation and provide useful marker to evaluate immune activation levels. In this study, we measured the surface expression of HLA-DR and CD38 and the DNA replication marker Ki-67, expressed intracellularly in PBMC and tissue MNC and these analyses are reported in Fig 4 and Fig 5 as group averages and in S2 Fig and S3 Fig for individual animals. Percentages of HLA-DR^+^/CD38^+^ cells were significantly lower in CD4^+^ and CD8^+^ T cells of the groups treated with ART plus p38 MAPK inhibitor compared to those treated with ART alone, whether treatment started at week 1 (p = 0.03, p = 0.009, for CD4 and CD8, respectively) or 6 post-infection (p = 0.002, p = 0.003, for CD4 and CD8, respectively), when the areas under the curve of the plotted parameters were compared in pair groups for the entire duration of the treatment (week 8 to week 60) (Fig 4A and 4B). The group receiving ART treatment since week 1 post-infection plus PH-797804 achieved the lowest frequency of immune activation markers in CD4^+^ and CD8^+^ T cells, although values did not return to baseline and remain approximately 2-fold higher. A similar trend was observed for the Ki-67 marker, which identifies activated cells, undergoing DNA synthesis and cell duplication (Fig 4C and 4D). When PH-797804 was used alone, levels of immune activation were no different than those observed in the control group, suggesting that the level of immune activation in these animals could not be impacted by the PH-797804 dose used here.

When the same analysis was carried out in in lymph node MNC (Fig 5A–5D), we found that, when combined with ART, the inhibitor impact was more significant in the animals initiating ART at week 6 and not as much in those initiating ART at week 1, where only the difference in CD38^+^/HLA-DR^+^ CD4 T-cell percentage was statistically significant, possible because the number of biopsy samples available for analysis (5) was smaller than for PBMC (Fig 4). The same analyses carried out in rectal MNC revealed that, when combined with ART, the inhibitor impact was significant in three of the four measured parameters in the animals initiating ART at week 1 but not in animals treated since week 6 post-infection, when AUCs were compared (Fig 5E–5H). However, differences in rectal CD38^+^/HLA-DR^+^ and Ki67^+^ CD8^+^ T cells between Group 3 and 4 were significant at 4 of 5 and 3 of 5 time points, respectively, when time point values were individually analyzed. Interestingly, blood and lymph node immune activation parameters of Group 4 (ART+ p38 inhibitor initiated in chronic infection, green lines) were comparable to those observed in Group 5 (ART only, initiated in acute infection, pink lines) (p = ns), supporting a significant benefit of this combination treatment when initiated in chronic infection. This result, although limited to phenotypic markers, is important, considering that ART is rarely initiated in the acute infection phase in HIV^+^ individuals and more commonly initiated during the chronic phase.

### Impact of PH-797804 on inflammatory cytokines in plasma and in T cells

**Fig 6:**
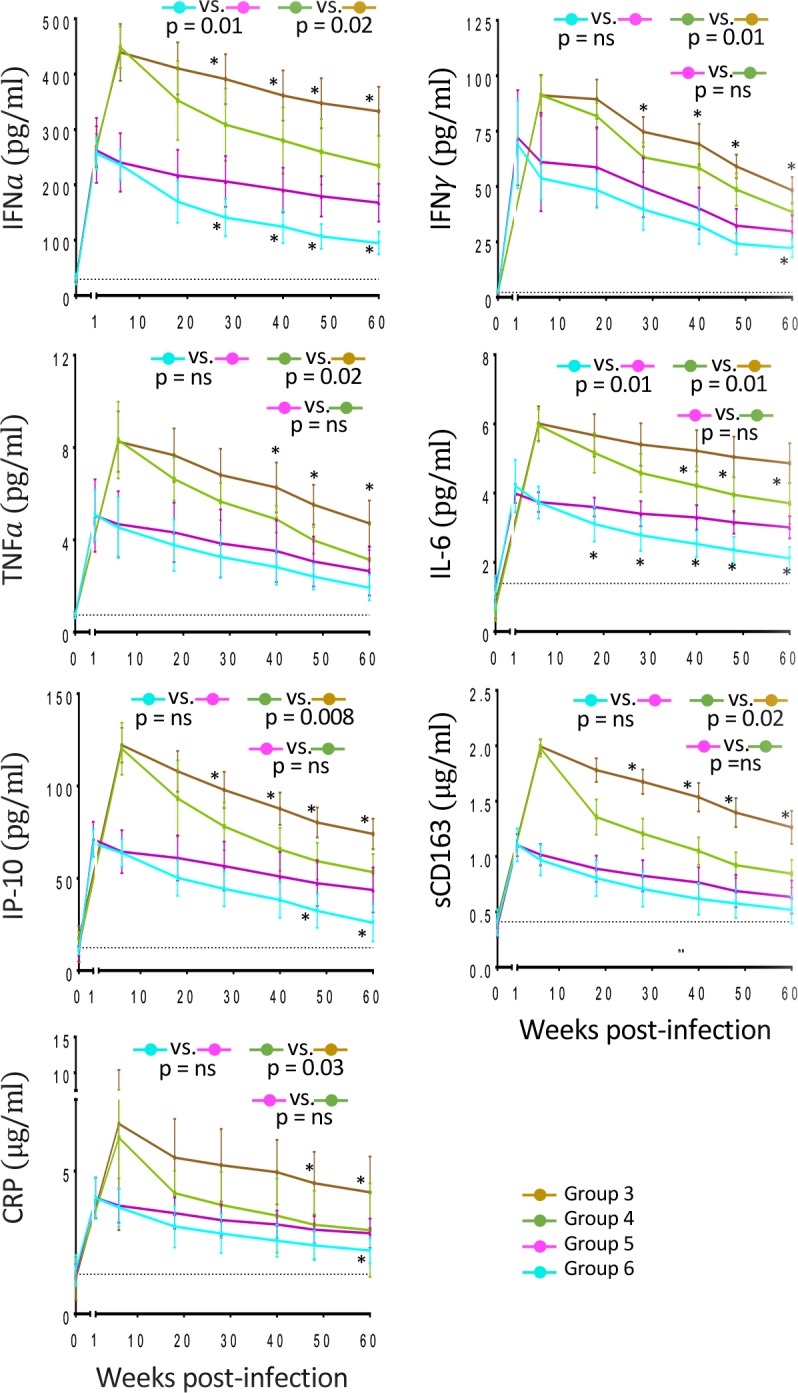
PH-797804 treatment reduces inflammatory cytokines and markers in plasma of SIV-infected RMs. ELISA longitudinal assessment of inflammatory cytokines levels in plasma of IFNα, IFNγ, TNFα, IL-6, IP-10 (pg/ml) and inflammatory markers CRP and sCD163 (μg/ml). Data are represented as mean ± SEM. The reported p values were calculated for the comparisons of the AUC from week 18 (first available time point after beginning of PH-797804 treatment) to 60 and refer to AUC comparisons in Group 3 vs. Group 4 and Group 5 vs. Group 6.

During lentiviral infection, the production of various inflammatory cytokines and biomarkers is known to be significantly higher than in normal subjects [62–65]. The levels of these cytokines, measured in plasma of SIV-infected RM by ELISA, were highest at peak viremia and were reduced during ART or ART plus PH-797804 treatment. The level of IFNα, IFNγ, TNFα, IL-6, IP-10, sCD163, a molecule shed by monocytes as a consequence of immune activation [66, 67], and C-reactive protein (CRP) were lower in the animals treated with ART and PH-797804 and the difference was statistically significant when Group 4 was compared to Group 3 (Fig 6 for group averages and S4 Fig for individual animals). Interestingly, even in this analysis, levels of IFNγ, TNFα, IP-10, sCD163 and CRP in Group 4 became comparable to those of Group 5, which initiated ART one week post-infection (p = ns). Of note is the fact that IFNα, although impacted by both ART and ART+PH-797804, remained with IFNγ the most abundant measured cytokine when compared to pre-infection values, despite undetectable viremia in some of the animals, suggesting an ongoing stimulation of innate responses. As a consequence, it is unlikely that activation of the numerous ISG, not covered here, can be fully controlled by simply inhibiting p38 MAPK, as it is highly likely that levels of ISG expression correlate with the levels of IFNs, which, although reduced, were still abnormal in this setting and not only influenced by p38 MAPK.

**Fig 7:**
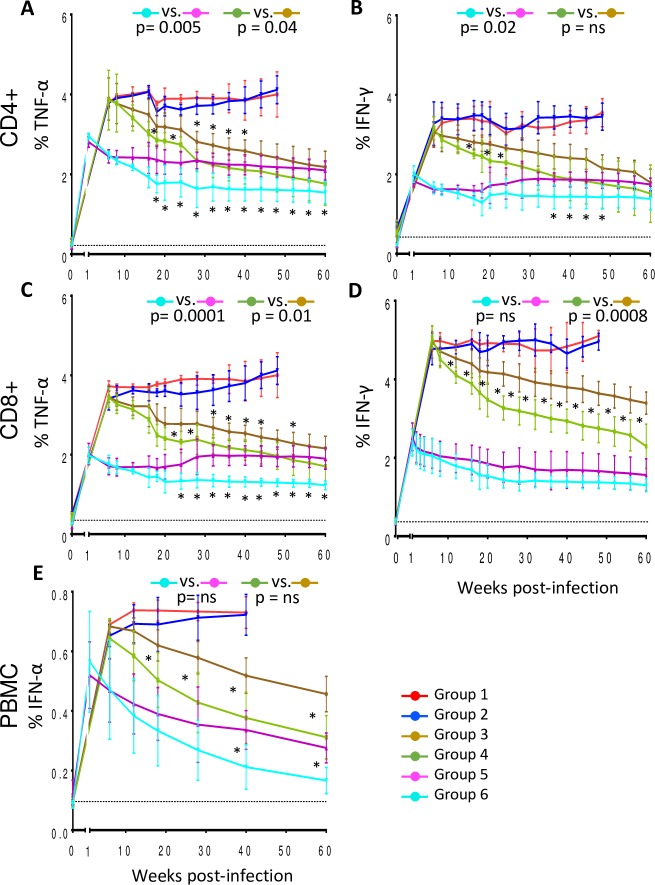
Inflammatory cytokine expression in CD4^+^ and CD8^+^ T cells of treated, SIV-infected RMs. Longitudinal analyses of frequency of CD4+ T cells expressing TNFα (A) and IFNγ (B) and of CD8^+^ T cells expressing TNFα (C), IFNγ (D), as detected in unstimulated, fresh PBMC obtained from animals after bleeding. E. Percentages of INFα^+^ cells in total PBMC. Data are reported as group mean ± SEM. The black, dotted line indicates the average of all 32 individual animal values measured before infection. The reported p values were calculated for the comparisons of the AUC from week 8 (first available time point after beginning of PH-797804 treatment) to 60 and refer to AUC comparisons in Group 3 vs. Group 4 and Group 5 vs. Group 6.

Plasma cytokine reduction was mirrored by reduction of percentages of T cells producing some of these cytokines (Fig 7 and S5 Fig). We found that the percentage of CD4^+^ T cells producing IFNγ and TNFα were significantly reduced in the groups receiving ART plus PH-797804 compared to ART alone, whether the treatment is started on week 1 (p = 0.005 for TNFα and p = 0.02 for IFNγ) or week 6 post-infection (p = 0.04 for TNFα), (Fig 7A and 7B). The percentages of TNFα^+^/CD8^+^ T cells in treated animals showed a significant reduction when the PH-797804 was added to ART, regardless of time of ART initiation (p = 0.0001 and p = 0.01 for comparisons between Group 5 and 6 and Group 3 and 4, respectively), while instead differences in the percentages of IFNγ^+^/CD8^+^ T cells were significant when Group 3 was compared to Group 4 (p = 0.0008) (Fig 7C and 7D) but not when Group 5 was compared to Group 6. In addition, the percentage of PBMC expressing IFNα was also significantly reduced when values of individual time points in Group 4 were compared to those in Group 3 (Fig 7E, asterisks) but this significance did not extend to the AUC comparative analysis. Taken together these data show that the administration of PH-797804 with ART reduced more significantly than ART alone the production of inflammatory cytokines and that, when added to ART in the chronic phase of the infection, which is the most common occurrence in HIV^+^ individuals, restored some parameters to the levels observed when ART was initiated in the acute phase, one week after infection.

### Treatment impact on preservation of immune system

**Fig 8:**
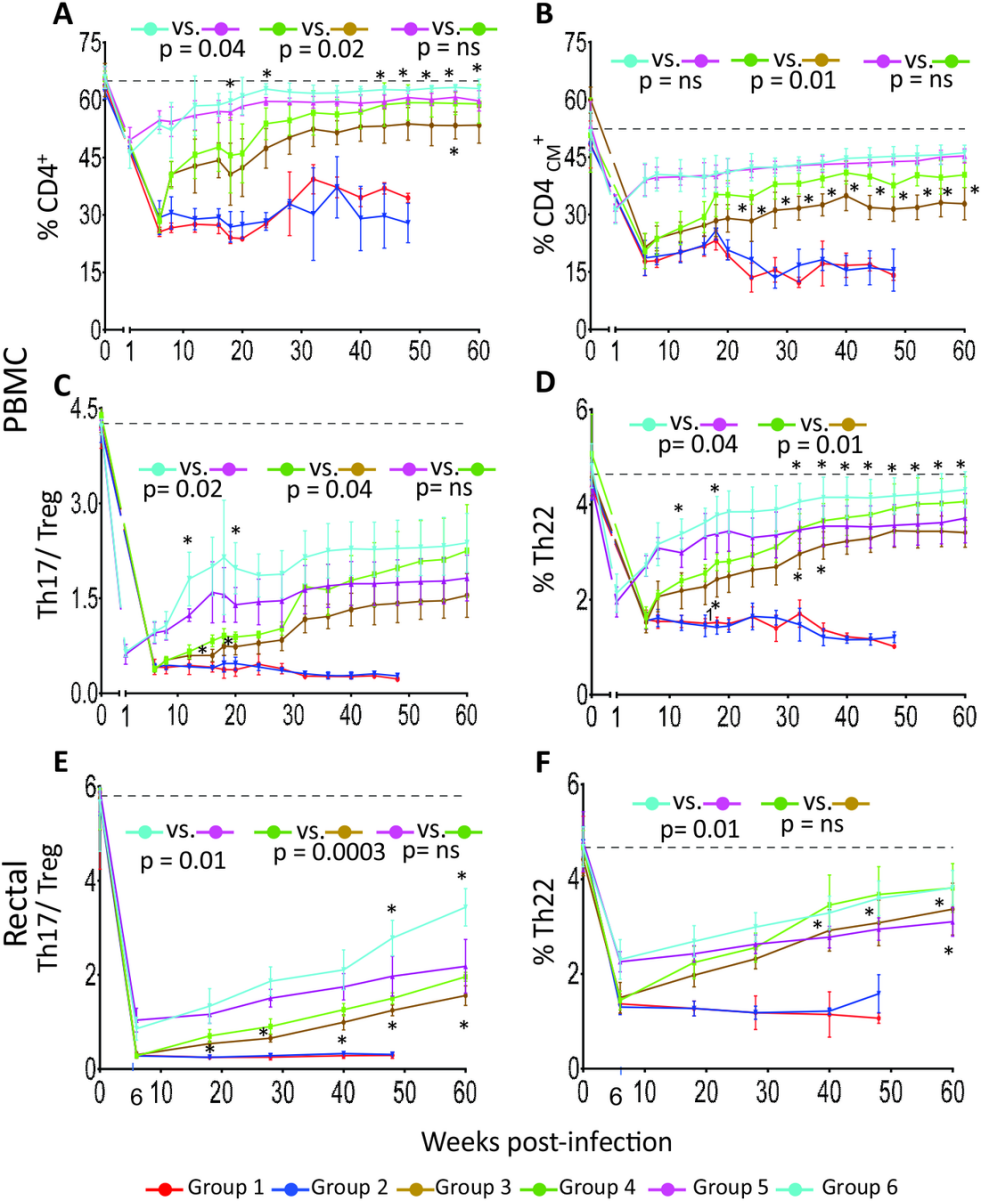
Recovery of immune system parameters during ART plus PH-797804 in SIV infected RMs. Longitudinal assessment of percentages of CD4^+^ T cells as a percentage of live CD3^+^ T cells (A) and of CD4_CM_^+^ T cells as a percentage of total CD4^+^ T (B). Longitudinal assessment of Th17/Treg ratio in PBMC (C) and in rectal MNC (E) and of frequency of CD4+ T cells producing IL-22 in PBMC (D) and in rectal MNC (F). Accumulation of IL-17 and IL-22 in CD4^+^ T cells was analyzed after PMA and Ionomycin stimulation. Data are represented as mean ± SEM. The black, dotted line indicates the average of all 32 individual animal values measured before infection. The reported p values were calculated for the comparisons of the AUC from week 8 to 60 for PBMC and from week 18 to 60 for rectal MNC and refer to AUC comparisons in Group 3 vs. Group 4 and Group 5 vs. Group 6.

The hypothesis we tested by adding a p38 MAPK inhibitor to ART was that, if immune activation contributes to the immune system deterioration, reduction of immune activation should result in preservation of immune cells. We evaluated the effect of PH-797804 treatment on percentages of total CD4^+^ T cells and C_M_ CD4^+^ T cells, which is considered an earlier prognostic marker in the infection, as C_M_ CD4^+^ T cells decline earlier than total CD4^+^ T cells [68]. The percentages of CD4^+^ T cells and C_M_ CD4^+^ T cells were significantly higher in Group 4 (ART + PH-797804 since week 6) compared to Group 3 (ART alone since week 6) (p = 0.02 for CD4^+^ T cells and p = 0.01 for C_M_ CD4^+^ T cells) (Fig 8A and 8B) and differences were not significant when Group 4 was compared to Group 5 (ART since week 1 post-infection). The same comparisons for Groups 5 and 6, receiving ART since week 1 post-infection, with CD4^+^ T-cell loss not as pronounced as in the groups initiating ART in the chronic phase, were significant for CD4^+^ T cells but not for C_M_ CD4^+^ T cells. This result supports the possibility that the addition of PH-797804 to ART permits a more significant recovery of CD4^+^ T-cell counts than ART alone when the virus damage of the immune system has been more severe and the combined regimen can compensate for a later initiation of ART.

Multiple studies have suggested that losses of intestinal Th17 and Th22 cells play a critical role in establishing intestinal mucosal immune dysfunction and are associated with the chronic immune activation typical of pathogenic HIV/SIV infections [69–80]. Several studies have reported that reciprocal changes in Th17 cells and Tregs occur during HIV and SIV infections and that the relative balance of Th17 and Treg subsets, expressed as a ratio of Th17 and Treg percentages, provides a prognostic index of disease progression more significant than each percentage considered individually [81–83]. In addition, Th22 cells play an important role in promoting innate immune defenses against bacterial and fungal infections in mucosal tissues, and in maintaining mucosal barrier integrity via mucus production and repair of damaged mucosal tissue [74–78, 80]. Therefore, we measured the impact of PH-797804 treatment on levels of the Th17 and Th22 CD4^+^ T-cell populations by evaluating their percentages in PBMC with intracellular staining in PBMC and in rectal MNC. We found that SIV infection reduced the Th17/Treg ratio but treatment improved it. The improvement was more significant in Groups 4 and 6, both receiving ART+ PH-797804, compared to Groups 3 and 5 (ART alone) (p = 0.02 for Groups 5 and 6 and p = 0.04 for Groups 3 and 4) (Fig 8C). We also evaluated Th17/ Treg CD4^+^ T-cell ratio and IL22^+^CD4^+^ T-cell percentage in the intestinal compartment, where loss of Th17^+^ and Th22^+^ cells during SIV infection is significant, and confirmed in rectal MNC an improved ratio in the groups receiving ART+ PH-797804 (p = 0.01 for Groups 5 and 6 and p = 0.0003 for Groups 3 and 4) (Fig 8E). A more significant recovery of PBMC Th22^+^ cells was observed when the inhibitor was administered (p = 0.04, Group 6 vs. 5, p = 0.01 Group 4 vs. 3, Fig 8D). Recovery of rectal Th22 CD4^+^ T cell percentages was significant for Group 6 compared to Group 5 (p = 0.01) while differences were not significant between Group 3 and 4, possibly due to limited samples and higher standard error for the groups, as percentages were higher in the group receiving PH-797804 and similar to those observed in Group 6 (Fig 8F). Taken together, these data indicate that the inhibition of T-cell activation achieved with PH-797804 treatment was significant enough to provide additional benefit to that observe with ART alone and positively impacted preservation or restoration of populations that are affected by chronic SIV infection. Importantly, the addition of the PH-797804 to ART initiated in the chronic phase resulted in immune population recoveries comparable than that observed when initiating ART in the acute phase of infection (compare data reported in green to those reported in pink), an event highly unlikely in most HIV infected individuals.

### Virus rebound after ART interruption

**Fig 9:**
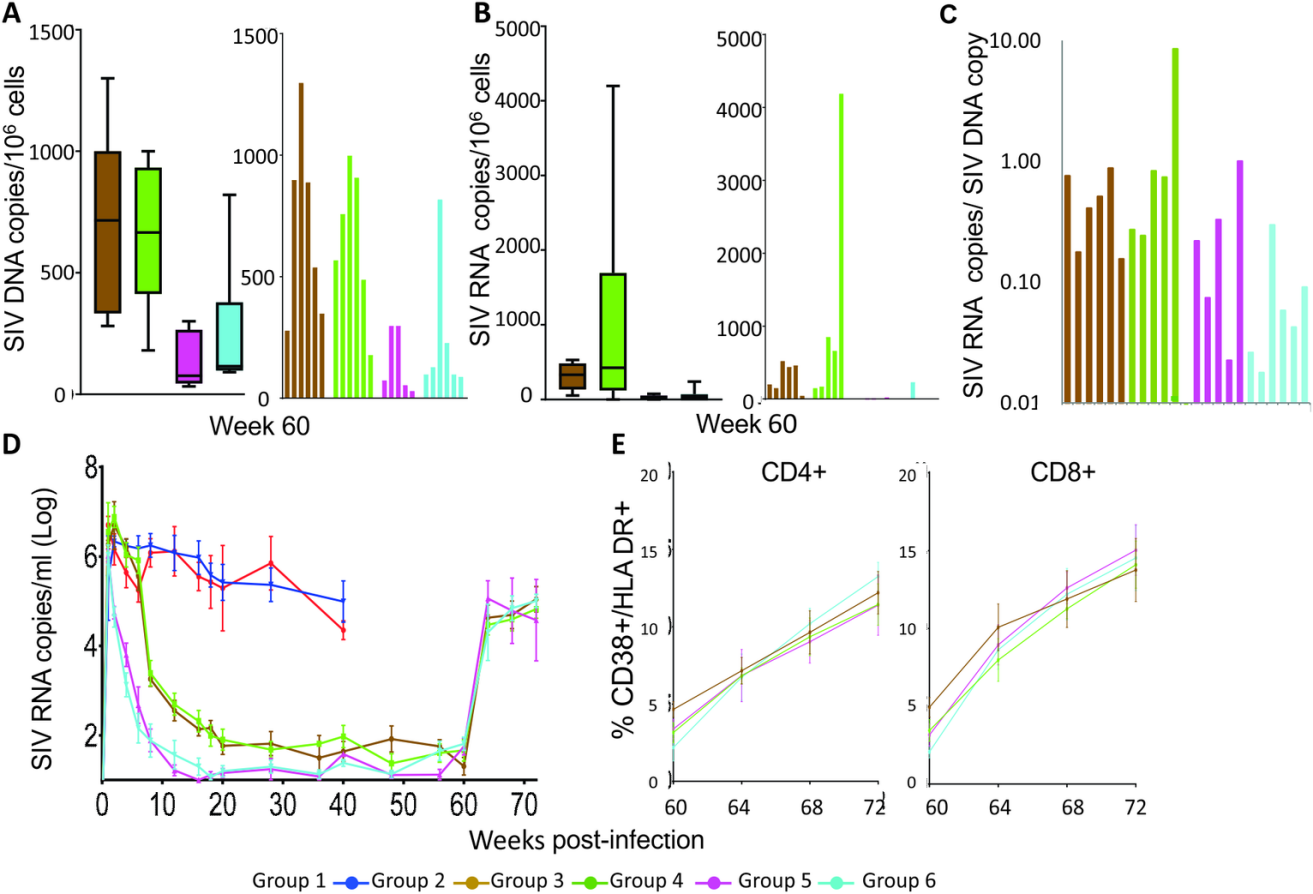
ART interruption, reservoirs and viral rebound. A. SIV DNA viral loads represented as median SIV DNA copy equiv./10^6^ cell equiv. (line), 1^st^ and 3^rd^ quartiles (box) and 10^th^ and 90^th^ percentile (whiskers) are shown. Numbers of SIV DNA copy equiv./10^6^ cell equiv. for each individual animal in the groups are shown in the second plot. B. SIV RNA viral loads represented as median SIV RNA copy equiv./10^6^ cell DNA equiv. (line), 1^st^ and 3^rd^ quartiles (box) and 10^th^ and 90^th^ percentile (whiskers) (first plot) are indicated. Numbers of SIV RNA copies/10^6^ cell DNA equiv. for each individual animal in the groups are shown in the second plot. C. Average number of SIV transcripts per SIV DNA+ cell in the four treated groups. D. Plasma viral loads are reported as RNA copies/ml. Level of detection for the assay is 15 copies/ml and undetectable levels are reported as 15 copies/ml. E. Percentages of CD38+/HLA-DR+ cells in CD4+ and CD8+ T-cell populations in blood, measured 4, 8, and 12 weeks post-ART interruption.

On week 60 after infection, ART and PH-797804 were interrupted. We investigated the viral burden in lymph node cells at the end of ART by evaluating DNA and RNA *gag* viral copies in MNC extracted from lymph nodes of animals in Group 3–6. We found that viral loads were significantly lower in Groups 5 and 6 that received ART since week 1 post-infection compared to Groups 3 and 4, where ART was initiated on week 6 post-infection (approximately 3.5 fold lower, p = 0.04), but not significantly different when the groups receiving the inhibitor were compared to the matched group that received ART alone. Similarly, the number of average SIV transcripts/10^6^ cells was lower in Group 5 and 6. When the total number of SIV RNA copies/ SIV DNA copies was calculated to obtain the average SIV genomic RNA transcripts /SIV infected cells, the number is very similar in all groups, ranging between 0.01 and 1 SIV *gag* RNA copies/ SIV DNA copy, except for one animal in Group 4, where the average is of 8.5 RNA copies/DNA copy (Fig 9A–9C). Averages below one SIV RNA copy / infected cell support the coexistence of SIV DNA+ cells without SIV transcripts, where the infection is latent, and others where number of SIV RNA molecules is higher than the calculated average. Single cell analysis is required to establish the fraction of cells in which transcription is active and quantitative infection assays to evaluate cells producing infectious virus. However, these results indicate that the level of suppression was similar and effective in all groups, compared well to those reported for suppressed HIV+ individuals and suppressed SIV+ macaques [84, 85], that the size of the reservoirs was established early on, and that the more prolonged viremia that occurs with later initiation of ART resulted in larger reservoirs. The addition of PH-797804 at week 6 post-infection did not impact the size of reservoirs, measured 54 weeks after inhibitor initiation. Despite the differences in total viral DNA burden in lymph nodes among groups, viremia measured for the first time after 4 weeks from ART interruption, rebounded to similar values in all animals, regardless of when ART was initiated or PH-797804 treatment was administered (Fig 9D). This rebound was comparable to that observed in HIV infection patients treated for a similar length of time, where 136 of 164 patients had significant and fast viral rebound after ART interruption [86, 87]. Not surprisingly, CD38^+^/HLA-DR^+^ percentages, which appears strictly linked to viral replication, rebounded as well and was indistinguishable among groups (Fig 9E). These data support the observation that reservoirs are established early in the infection and that initiating ART in the acute phase reduces but does not eliminate the establishment of reservoirs, possible because it takes time for ART to bring viremia to undetectable levels and reservoirs continue to be seeded for days after ART initiation [88–91]. Decay of reservoirs is time dependent and time to rebound is clearly affected by the duration of ART, which in this trial was restricted to 59 or 54 weeks and it can be significantly longer in ART-treated, HIV infected individuals, evaluated for the same virological parameters.

## Discussion

Immune activation is the differentiating feature between infection is species that progress to AIDS versus species that do not and persistent immune activation remains despite immunosuppressive ART treatment. ART treatment substantially reduces viral loads and, consequently, immune activation. However, CD8^+^ T-cell activation does not decrease proportionally to the decrease of viral loads and its levels have been inversely linked to the degree of CD4^+^ T cell reconstitution during ART [92]. HIV-associated pathology and, in particular, HIV associated neurological disorders (HAND), did not decrease after the introduction of ART in the same proportion that one would expect if only dependent on blood viral burden [93, 94]. Immune activation and/or ART toxicity have been postulated as possible explanations for this outcome. The causes of residual immune activation have been attributed to factors such as residual HIV replication, persistent microbial translocation, and viral co-infections such as CMV [references in [92]]. It seems therefore ideal to conceive a treatment that combines ART and inhibitors of immune activation. Only limited data are available for this approach, with focus on combination of ART with Cyclosporin A during acute/early infection for one short cycle [95–98]. A significant effect on the residual CD8^+^ T-cell immune activation was observed with this approach [97]. We investigated whether suppression of one of the key player in immune activation, p38 MAPK, can impact SIV-mediated immune activation during ART. We found that this treatment, when combined with ART, does positively impact virus-mediated immune activation and permits preservation of subpopulations that are significantly affected during the infection. However, reduction of immune activation did not appear to impact viral reservoirs, known to be established early on in SIV and HIV infections, even when ART was initiated just a few days after infection [91].

The addition of the p38 MAPK inhibitor to ART significantly suppressed multiple parameters of immune activation. The observed suppression was not complete and residual immune activation was approximately 50–65% of that observed in animals receiving ART alone. Only one inhibitor dose was explored and investigation of additional doses would be preferable. As it is for ART, the combination of multiple inhibitors of immune activation pathways, aimed at different targets linked to immune activation, may ultimately provide a more substantial control of immune activation during ART, considering that redundancy exists in the immune system and that p38 MAPK activity can also, in part, be carried out by other kinases like JNK and ERK. Indeed, when we carried out *in vitro* experiments, we found that features of immune activation that are observed after HIV infection of APC could most significantly be impacted by treatment with a p38 MAPK inhibitor but also by a JNK inhibitor and to a lesser extent by an ERK inhibitor [45–47].

p38 MAPK inhibition provided diverse benefits with improvement of multiple immune parameters and resulted in a significant preservation or better restauration of cell populations that are critical to the immune system (Fig 8). Recovery of percentages of total CD4^+^, C_M_ and Th22 CD4^+^ T cells, and of Th17/Treg ratio was more substantial in Group 4 that initiated ART and PH-797804 around the time when viremia reaches the set point (week 6) and when CD4^+^ T-cell depletion had reached values as low as those in the animals that remained untreated. These results support the possibility that the addition of PH-797804 permits a more significant recovery of CD4^+^ T-cell populations than ART alone, especially when the damage to the immune system has been more prolonged and substantial. In Group 4, recovery of affected cell populations reached levels comparable to those observed in Group 5 that initiated ART only one week after infection (Fig 8). The fact that the addition of PH-797804 provided a more noticeable benefit when ART was initiated later in the infection, when the viral damage to the immune system has been more severe, suggests that this treatment could be significant in HIV^+^ individuals, where initiation of ART after reaching viremia set-point is the more common occurrence than ART initiated during the acute stage. Lastly, significant reduction of both PD-1 percentages and MFI in CD8+ T-cells may permit a more effective anti-retroviral immune response. As only one PH-797804 dose was tested, the benefit could further increase if the ideal dose was identified.

IL-1β inhibition with canakinumab has recently been shown to reduce cardiovascular events in patients with coronary arterial disease and has also been shown to decrease immune activation in treated HIV patients, but there are concerns about its safety, particularly infectious complications [99, 100]. We did not detect reduced immune responses to SIV while treating the animals with the p38 MAPK inhibitor. The setting in which the p38 MAPK inhibitor was used (single caged macaques, kept indoor) significantly reduces the exposure to infectious agents and therefore may not provide a sufficient indicator of lack of impact on infection control. However, this inhibitor could be safer than an IL-1β inhibitor, as it is highly selective for p38 MAPK and does not inhibit the other two members of the family of major mitogen-activated protein kinases, JNK and ERK. These kinases are partially, even if not fully, overlapping with p38 MAPK function and, therefore, the inhibition of some pathways affected by p38 MAPK may not be absolute, avoiding their complete shutdown. This could also be a reason why the observed reduction of immune activation was limited, even if significant. Although a full analysis of ISG expression in blood and tissue populations is beyond the scope of the report, this investigation could reveal in more subtle details whether some ISG are more affected than others and provide additional information on the activity of PH-797804 *in vivo*. Such analysis will be object of future, larger studies. The expectation is that, if carried out in the setting of this study, a reduction of ISG that could mirror the reduced levels of circulating IFNα and IFNγ would be detected in animals treated with PH-797804 and ART, and that a more significant suppression of IFN pathways could only be achieved by interfering with other mitogen activated kinases that can overlap p38MAPK or their upstream regulators.

One expectation of this study was that by reducing immune activation, the availability of activated CD4^+^ T cells that are a preferred target of infection would also be reduced and therefore reservoir seeding could be impacted. However, we did not find significant differences in reservoir size when Group 3 was compared to Group 4 and Group 5 to Group 6. It is possible that differences in rebound time could have been observed if earlier sampling had been obtained, when Group 3 and 4 were compared to Group 5 and 6. As by week 4 all groups treated with ART, with or without PH-797804, had reached similar levels of viremia, this delay would have been limited and unlikely to impact the course of disease progression. This results also suggests that reducing the immune activation status did not translate in reduced reservoirs and supports the observation that viral reservoirs are set early on in the infection [89, 91], before the beginning of ART, are very long-lived [101], and not necessarily exclusively made of resting, infected cells. However, SIV DNA and RNA viral loads in lymph nodes did not increase because of treatment, suggesting that the antiviral effect of ISG was not impacted.

The fact that rebound occurs without administration of latency reactivating agents seems to support the possibility that reservoirs include cells that are not fully resting. Indeed, rebound is observed in HIV^+^ individuals without treatment of activating agents, although external immune activating stimuli could contribute to the occurrence. It is also possible that in our trial ART was carried out for a period that is relatively short compared to similar studies done in HIV^+^ individuals, who have received ART for many years, and that the physiological decay of the reservoirs established before the initiation of treatment was not as advanced, opening the possibility that a difference could be observed only if the animals were kept on ART plus PH-797804 for a longer time.

An alternative possibility is that the immune activation detected during ART suppression is not fully independent of viral gene expression but stems from continuous, low-level virion production in tissues that maintains TLR activation. As HIV transcription and SIV entry inhibitors were not part of the regimen of this trial, the therapy administered could permit partial rounds of the virus life cycle, with release and cell entry of non-infectious virions, even if productive infection cannot be achieved after entry. However, entry or uptake of non-infectious virions could be sufficient to trigger TLR signaling, even in the absence of reverse transcription and integration, and this activation could support persistent immune activation. If complete suppression of SIV antigen production were achieved and viral genomes were truly latent during ART, one does not explain the fact that CD8^+^ T-cell depletion leads to viremia rebound while ART is still in place in SIV-infected RM, as CD8^+^ T-cell activity requires antigen production to be effective. Evaluation of TLR-activated pathways in the context of ART alone and with immune activation suppression is an important goal of future studies. The possibility of persistent, low level antigenemia, which would offer a significant additional source for persistent immune activation, is not sufficiently considered and explored and requires further investigation.

## Materials and methods

### Ethics statement

The study received institutional review board approval at the Tulane Primate Research Center, where the macaques used in the study were housed. IACUC approval number of the study is: P0236R. Animal care methods are consistent with the recommendations of the panel on euthanasia of the American Veterinary Medical Association. This study was also carried out in strict accordance with the recommendations in the “Guide for the Care and Use of Laboratory Animals of the National Institutes of Health, National Academy Press, 1996) and with the recommendations of the Weatherall report: “The use of non-human primates in research”. The institution also accepts as mandatory the PHS “Policy on Humane care and use of Laboratory Animals by Awardee Institutions” and the NIH “Principles for the Utilization and Care of Vertebrate Animals used in Testing, Research and Training”.

1. Experimental design. This investigation is designed to extend our previous studies by investigating approaches to reduced immune activation in Rhesus macaques using a drug that inhibits p38 MAPK, a cellular protein activated by HIV and SIV and linked to immune activation. During the course of these studies a number of clinical procedures were performed. All procedures were performed under anesthesia using ketamine, and all efforts were made to minimize stress, improve housing conditions, and animals were provided with enrichment opportunities including objects to manipulate in the cages, varied food supplements, foraging and task-oriented feeding methods, and interaction with caregivers and research staff.
  - Viral inoculation: Animals were anesthetized with ketamine HCl 10-15mg/kg IM and inoculated with high dose SIV_mac251_ i.v.
  - Physical examination: Animals were weighed and receive a complete physical exam each time they are anesthetized for a clinical procedure or at least monthly.
  - Phlebotomy: Phlebotomy was performed under ketamine 10-15mg/kg IM through the femoral vein by standard techniques. 2.5mls of EDTA and 2.5mls of whole blood were obtained at each time point. Blood was utilized for determination of viral load by quantitative PCR, determination of lymphocyte subsets and evaluation of circulating cytokine levels.
  - Collection of colorectal and lymph node biopsies. If needed, biopsies were obtained from the large intestine (colon, rectum) using sterile forceps and a small pinch biopsy device (Olympus endoscopic biopsy forceps) inserted 4–8 cm into the rectum. Animals were anesthetized with telazol and were given 0.005mg/kg buprenex after this procedure and again that evening. Physical examination, with visual inspection of rectal mucosal surfaces, was performed if discomfort is observed after 48 hours. Appropriate antibiotics were administered if an infection is detected. Inguinal and axillary lymph node biopsies were also collected.
2. Justification. The need to use non-human primates in these studies is justified by the risks and uncertain benefit of carrying out trials of vaccines for AIDS in humans. SIV-infected macaques are acknowledged to represent the best animal model for the study and development of AIDS vaccines. In vitro models cannot reproduce the complex interplay between the host’s immune response and viral infection. Rhesus macaques are susceptible to AIDS upon infection with SIV and have an immune system similar to man. There are no other species that can be infected with SIV and therefore vaccine and protection studies cannot be carried out in other primate or non-primate species. These studies propose the absolute minimum number of animals required to carry out a pilot testing of the drug.
3. Veterinary care. The Tulane Primate Center animal management program is accredited by the Association For Assessment and Accreditation of Laboratory Animal Care (AAALAC #000594) and meets National Institutes of Health standards as set forth in “The Guide for the Care and Use of Laboratory Animals” (National Academy Press, 1996). There is on file with the Office of Protection from Research Risks an approved Assurance of Compliance. In vivo animal work was carried out at the Tulane Primate Research Center. The Center is located 35 miles north of Tulane University New Orleans campus and currently houses 5,000 non-human primates including 534 SIV-infected rhesus macaques on infectious disease studies. The Center consist of seven BSL2 containment facilities and two BSL3 containment facilities including a Regional Biosafety 2 Laboratory. These facilities are equipped with dedicated clinical procedure areas, surgery suites and necropsy room and contain state-of-the art clinical equipment of management immunodeficient animals. The Division of Primate Resources provides, coordinates, and administers all veterinary and animal husbandry services at Tulane Primate Research Center. Dr. Rudolf P Bohm is chairman of the division and directs all aspects of the division. Animals housed in the biocontainment facilities receive a daily health check by both animal care technicians and veterinary professional staff. The clinical veterinary that supervised and carried out the work described in this article has work at this facility for seven years. All animals received a complete physical examination, complete blood count and chemistry panel once every three months prior to inoculation and monthly following inoculation. In addition to the procedures above, CD4 T lymphocyte count and viral load are determined for SIV-infected animals and used for clinical assessment. In the event that clinical disease is recognized, the attending veterinarian and principal investigator are notified. The animal is examined, the medical record reviewed and laboratory work repeated if needed. A plan of action is agreed upon between the veterinarian and principal investigator that may involve continued observation, treatment, or euthanasia of the animal. All animals housed in the biocontainment facility have strict criteria for euthanasia, which are determined prior to initiation of experiments through consultation between the attending veterinarians, principal investigators and members of the Tulane Primate Research Center IACUC. The Tulane Primate Research Center has developed a comprehensive environmental enrichment and psychological well-being plan for primates, which is available for inspection by the United States Animal and Plant Health Inspection Service (APHIS) and to officials of any pertinent funding agency. The Tulane Primate Research Center Standing Committee on Animals (IACUC) must approve an investigator's request for exemption of animals from these plans. In addition, the attending veterinarian may exempt individual animals for health-related reasons. The IACUC documents Tulane Primate Research Center compliance with the plan during semiannual facility inspections. Animals in this study were purchased from the Center’s Specific pathogen free breeding colony and housed in the Tulane Primate Research Center biocontainment facilities. These facilities meet all guidelines as set forth by the CDC/NIH in Biosafety in Microbiological and Biomedical Laboratories 4th edition. A total of 32 animals were studied over a four-year period to evaluate the effect of treatment with a p38 MAPK inhibitor on SIV-mediated immune activation and on viral loads and possibly protection from AIDS development. Following selection animals were moved to the Tulane Primate Research Center biocontainment facilities, acclimatized and base line measurements made. Animals were then randomly assigned to control or treatment groups and infected by the i.v. route. Animals were followed prospectively through sequential blood draws, rectal biopsies and lymph node biopsies to evaluate immune responses. The clinical staff at the Tulane Primate Research Center routinely performs these procedures. Following infection with SIV_mac251_, animals were treated with ART and/or the p38 inhibitor, and blood obtained for CBC, lymphocyte subset analysis, antiviral immune responses, markers of immune activation in monocytes and T cells.
4. Procedures for ensuring that discomfort, pain and injury is limited: SIV_mac251_ is a pathogenic virus that causes progressive depletion of CD4^+^ T lymphocytes and AIDS. Mean survival is approximately 18 months following intravenous challenge. Some animals in these experiments may be protected from disease following drug treatment. Control animals and unprotected animals were likely to develop AIDS and opportunistic infections. When warranted, they received standard clinical care including but not limited to analgesics (buprenex 0.005mg/kg IM BID), intravenous fluids, antibiotics and other supportive therapy. If animals developed AIDS, euthanasia was performed based on the following criteria:
  - Weight loss >15% in 2 weeks or >30% in 2 months
  - Documented opportunistic infection
  - Persistent anorexia >3 days without explicable cause
  - Severe, intractable diarrhea
  - Progressive neurologic signs
  - Significant cardiac and/or pulmonary signs
  - Loss of CD4+ T cells below 200 or 10%
  - Any other serious illness
5. Euthanasia. Euthanasia was performed when AIDS was diagnosed or at the closing of the experiment (approximately 1.4 years after infection). A necropsy was performed. Tissues were evaluated to determine the extent of lymphoid organ demise. Euthanasia was performed following induction of anesthesia with 15mg/kg ketamine HCl. >>50mg/kg of sodium pentobarbital was be administered intravenously. These methods are consistent with the recommendations of the panel on euthanasia of the American Veterinary Medical Association."

### Animals, SIV infection and interventions

Thirty-two male RM (*Macaca mulatta*), ranging in age between 2.40 and 4.33 years when the study was initiated, were included in this study and were housed at Tulane Primate Research Center, Tulane University, Covington, Los Angeles. Animals were evaluated for the expression of the following MHC molecules: A*01, A*02, A*08, A*11, B*01, B*03, B*04, B*08, B*17. None of the animals included in the study tested positive for the protective alleles A*01, B*08, and B*17 [102, 103]. Animals were divided in 6 groups and infected intravenously (i.v.) with 10 TCID50 SIVmac_251_ (day 0). Groups 1 and 2 included four animals, Groups 3 to 6 included six animals. The animals in Group 1 were left untreated as control. Since week 6 after infection, Group 2 received PH-797804 alone, group 3 initiated antiretroviral therapy (ART) and group 4 received ART and PH-797804. Group 5 initiated ART one week post- infection as did Group 6, who also received PH-797804 starting from week 6 post-infection. ART consisted of two reverse transcriptase (RT) inhibitors, tenofovir (PMPA, 20 mg/kg, and emtricitabine (FTC, 30 mg/kg), and the integrase inhibitor dolutegravir (DTG, 2.5mg/kg s.i.d), all administered i.m. [104]. The animals in group 2, 4 and 6 received 3 cycles of PH-797804 (10 mg s.i.d, orally administered), each of 12 weeks (6–18, 28–40 and 48–60 weeks). ART and PH-797804 treatment were interrupted on week 60 and animals were monitored monthly for virus rebound until week 72 post infection.

### Sample collection and processing

Blood were collected at various time points, approximately every 4 weeks; rectal and lymph node (LN) tissues were biopsied before infection and at the beginning and end of PH-797804 cycles. Briefly, after Telazol anesthesia, seven to eight biopsies/animal/time points were obtained from the rectum and blood was collected in EDTA. PBMC and plasma were separated using Ficoll-Hypaque gradient centrifugation. Rectal and lymph node biopsy-derived MNC were isolated by digestion with 1 mg/ml collagenase for 1 h at 37°C, passed through a 70-mm cell strainer to remove residual tissue fragments and separated using Ficoll-Hypaque gradient centrifugation [105].

#### Viral load evaluation

Plasma SIV RNA levels were measured by real-time RT-PCR assay as described [106]. The Lifson assay has a threshold sensitivity of 15 copy equivalents per milliliter. Inter-assay variation is <25% (coefficient of variation). Mean viral loads were calculated by transforming the number in its logarithmic value and averaging the logarithmic values of all the animals of the group at one specific time point [106]. Evaluation of SIV DNA and RNA viral loads in lymph node cells was carried out using primers for a conserved SIV *gag* region, according to the methods published in [84].

### Polychromatic flow cytometric analyses

Polychromatic flowcytometric analysis was carried out in PBMC, LN and rectal biopsy MNC according to standard procedures for membrane and intracellular staining, using a panel of mAbs (see below) shown to be cross reactive with RMs [105]. The percentages of CD4^+^ T cells and C_M_ CD4^+^ T cells, immune activated CD4^+^ and CD8^+^ cells, FOXP3+ T regulatory cells, IFNγ^+^ and TNFα^+^ CD4^+^ and CD8^+^ T cells, and IP-10^+^, pSTAT1^+^ and IRF7^+^ PBMC were evaluated in unstimulated cells by membrane and intracellular staining (ICS) [107] and reported as the percentage of CD4^+^, CD8^+^ T cells, or PBMC that express one or more markers. Accumulation of IL-17 and IL-22 in CD4^+^ T cells was analyzed after phorbol 12-miristate-13 acetate (PMA,10 mg/ml) and Ionomycin (1mg/ml) stimulation, using the same technique. Briefly, aliquots of PBMC, LN and rectal MNC were re-suspended at 10^6^ cells/ml in medium with or without stimulation and containing Golgi stop (BD Bioscience, San Diego, CA) and incubated at 37°C for ~ 12 hr. The cells were then washed and stained with the appropriate panel of antibodies for 30 minutes in the dark at room temperature, followed by fixation and permeabilization. After permeabilization, the cells were stained intracellularly with monoclonal antibodies against the cytokines of interest for 1 hour in the dark. Data were acquired on BD LSR II flow cytometer using FACSDIVA software. After acquisition, data were analyzed using the FlowJo software.

The following anti-human, macaque cross-reacting, or anti-macaque antibodies were used in this study: anti-CD3-pacific blue/PerCp-Cy5.5 (clone SP34-2), anti-CD4-Amcyan (clone L200, anti-CD8-APC-Cy7 (clone RPA-T8), anti-Ki-67-Alexa Fluor-700 (clone B56), anti-HLADR-PE-CF594 (clone G46-6), anti-TNFα- PE (clone MAb11), anti-IFNγ-Alexa Fluor-700 (clone B27), anti-IRF7-APC (clone K47-671), anti- pSTAT1-Pacific blue (clone 14/ pSTAT1), anti-CD14-Pe-Cy7 (clone M5E2) (all from BD Pharmigen); anti-FOXP3-FITC (clone 206D), anti-IP-10-PE (clone J034D6), anti-CD28-Pe-Cy5 (clone CD28.2), anti-CD95-FITC (clone DX2), anti-PD-1-PerCp-Cy5.5 (clone EH12.2H7), anti-IL-2-APC (clone MQ1-17H12) (all from BioLegend, San Diego, CA); anti-IL-17- PerCp-Cy5.5 (clone- eBio64DEC17), anti-IL-22-APC (clone IL22JOP) (both from eBiosciences, San Diego, CA) and anti-CD38-APC (clone OKT10) from NHP Reagent Resource, Boston, Massachusetts).

### Plasma levels of inflammatory markers

The levels of IFNγ, TNFα, IL-6, and IP-10 in plasma were measured using commercially available ELISA kits from U-CyTech, Utrecht, Netherlands, according to manufacturer’s instructions. CRP, sCD163 and IFNα plasma levels were measured using a monkey CRP ELISA kit (Life Diagnostics Inc., West Chester, PA), a sCD163 ELISA kit (My Biosource, CA, USA) and an IFN α ELISA kit (PBL Assay Science, NJ, USA).

### Statistics

Calculations and statistical analyses were performed using the GraphPad Prism version 7 software. Normality distribution values were calculated using the D'Agostino-Pearson omnibus test. When values were normally distributed, p values were calculated using unpaired t-test, when non-normal, Wilcoxon-Mann-Whitney (rank sum) test was applied. Between-group comparisons at individual time points were carried out with Wilcoxon-Mann-Whitney (rank sum) test or unpaired using t-test depending on population normality values. AUC analyses were carried out by calculating an AUC for the time course values of each animal and areas in one group were compared to those is a second group. Results of statistical analyses were considered significant if they produced *p* values ≤ 0.05.

#### Study approval

The study received institutional review board approval at the Tulane Primate Research center, where the macaques used in the study were housed. IACUC approval number of the study is: P0236R.

## Supporting information

S1 Fig Evaluation of p38 MAPK inhibitor treatment efficacy. **A.** Gating strategy for ICS to evaluate percentages of IRF-7, pSTAT1 and IP-10 in PBMC. Columns report analyses for: 1. Singlets; staining for: 2. Live/Dead, 3. IRF7, 4. pSTAT1, 5. IP-10. First row shows fluorescence minus one staining (FMO) in one representative sample for IRF7, pSTAT1, IP-10. Absence of the antibody indicated above was used to position gates to evaluate percentage of positive cells for that specific antibody. Rows 2–5 show staining for IRF7, pSTAT1, IP-10 in one representative sample for each group. B. Table report average group percentages of PBMC subpopulations on week 6 when inhibitor therapy was initiated and on week 60 when it was stopped. Differences were minimal between paired groups (Group 3 and 4 and Groups 5 and 6). C. Percentages of CD4^+^ and CD8^+^ T cells in PBMC expressing pSTAT1, IRF7, and IP-10. D. Percentages of CD4^+^ and CD8^+^ T cells in lymph node MNC expressing pSTAT1, IRF7, and IP-10. The black, dotted line indicates the average of the 32 animal values measured in samples obtained on the day before infection. The reported p values were calculated for the comparison of the AUC from the first time point available after p38 MAPK inhibitor treatment initiation to 60 and refer to AUC comparisons in paired groups. Between group comparisons at individual time points were carried out with Wilcoxon-Mann-Whitney (rank sum) test. Asterisks mark significant time point comparisons for Group 3 vs. Group 4 (asterisks above brown line) or Group 5 vs. Group 6 (asterisks below blue line).
(PDF)

S2 Fig Longitudinal analysis of immune activation marker expression in PBMC T cells of SIV-infected and treated or untreated RMs. Percentages of HLA-DR^+^/CD38^+^ in CD4^+^ (A) and CD8^+^ (B) T cells and of Ki-67^+^ in CD4^+^ (C) and CD8^+^ (D) T cells in PBMC. Data are reported for each individual animal. The black, dotted line indicates the average of all 32 individual animal values measured before infection. The reported p values were calculated for comparisons of AUC between week 8 and 60 in paired groups.
(PDF)

S3 Fig Longitudinal analysis of immune activation marker expression in tissue T cells of SIV-infected and treated or untreated RMs. Data for lymph node and rectal tissue T-cell expression of immune activation markers in biopsies collected at each PH-797804 treatment cycle start and end time points are shown. Panels report percentages HLA-DR^+^/CD38^+^/CD4+ (A) or Ki-67^+^/CD4^+^ T cells (B) in inguinal lymph nodes and in rectal mucosa (E and F, respectively), percentage of HLA-DR^+^/CD38^+^/CD8^+^ (C) or Ki-67^+^/CD8^+^ T cells (D) in lymph nodes and in rectal mucosa (G and H, respectively). Data are represented for each individual animal. The black, dotted line indicates the average of all 32 individual animal values measured before infection. The reported p values were calculated for comparisons of AUC between week 18 (first available time point after beginning of PH-797804 treatment) to 60.
(PDF)

S4 Fig PH-797804 treatment reduces inflammatory cytokines and markers in plasma of SIV-infected RMs. Longitudinal assessment of inflammatory cytokines levels in plasma of IFNα, IFNγ, TNFα, IL-6, IP-10 (pg/ml) and inflammatory markers CRP and sCD163 (μg/ml) by ELISA. Data are represented for each individual animal. The reported p values were calculated for comparisons of AUC between week 18 and 60 in paired groups.
(PDF)

S5 Fig Inflammatory cytokine expression in CD4^+^ and CD8^+^ T cells of treated, SIV-infected RMs. Longitudinal analysis of frequency of CD4+ T cells expressing TNFα (A) and IFNγ (B) and of CD8^+^ T cells expressing TNFα (C), IFNγ (D), as detected in unstimulated, fresh PBMC obtained from animals after bleeding. E. Percentages of INFα^+^ cells in total PBMC. Data are reported for each individual animal. The black, dotted line indicates the average of all 32 individual animal values measured before infection. The reported p values were calculated for comparisons of AUC between week 8 and 60 in paired groups.
(PDF)
