# Supplementary figures and images for: Inhibition of p38 MAPK in combination with ART reduces SIV-induced immune activation and provides additional protection from immune system deterioration
Source: PLoS Pathog. 2018 Aug 30;14(8):e1007268. doi: 10.1371/journal.ppat.1007268 (PMC6135519; doi:10.1371/journal.ppat.1007268)

A

**IP-10**

| B       | Week 6    |           |           |          | Week 60    |           |          |           |
|---------|-----------|-----------|-----------|----------|------------|-----------|----------|-----------|
|         | % CD3     | % CD4     | % CD8     | % CD14   | % CD3      | % CD4     | % CD8    | % CD14    |
| Group 3 | 56.5±3.6  | 40.6±3.56 | 59.3±3.73 | 5.2±0.78 | 55.25±2.68 | 57.0±2.59 | 43.±2.53 | 5.75±0.8  |
| Group 4 | 53.9±2.64 | 42.4±3.75 | 58.5±3.65 | 5.9±0.54 | 56.2±2.27  | 58.9±2.05 | 41.9±2.1 | 5.51±0.65 |
| Group 5 | 54.7±2.82 | 53.3±2.5  | 47.6±2.67 | 5.4±0.46 | 55.8±3.23  | 59.8±2.34 | 40.2±2.4 | 5.62±0.72 |
| Group 6 | 55.6±4.2  | 56.1±2.94 | 44.8±3.1  | 5.6±0.56 | 57.5±2.45  | 63.0±1.87 | 37.±1.9  | 5.7±0.5   |

C

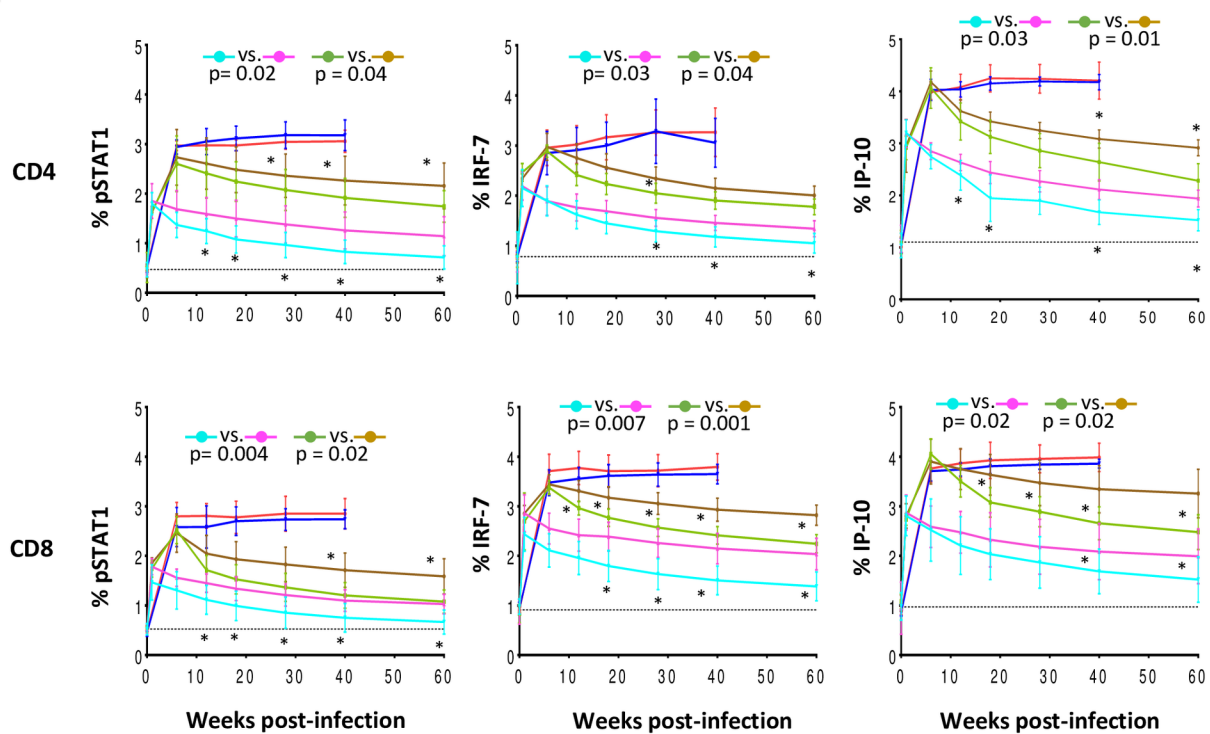

D

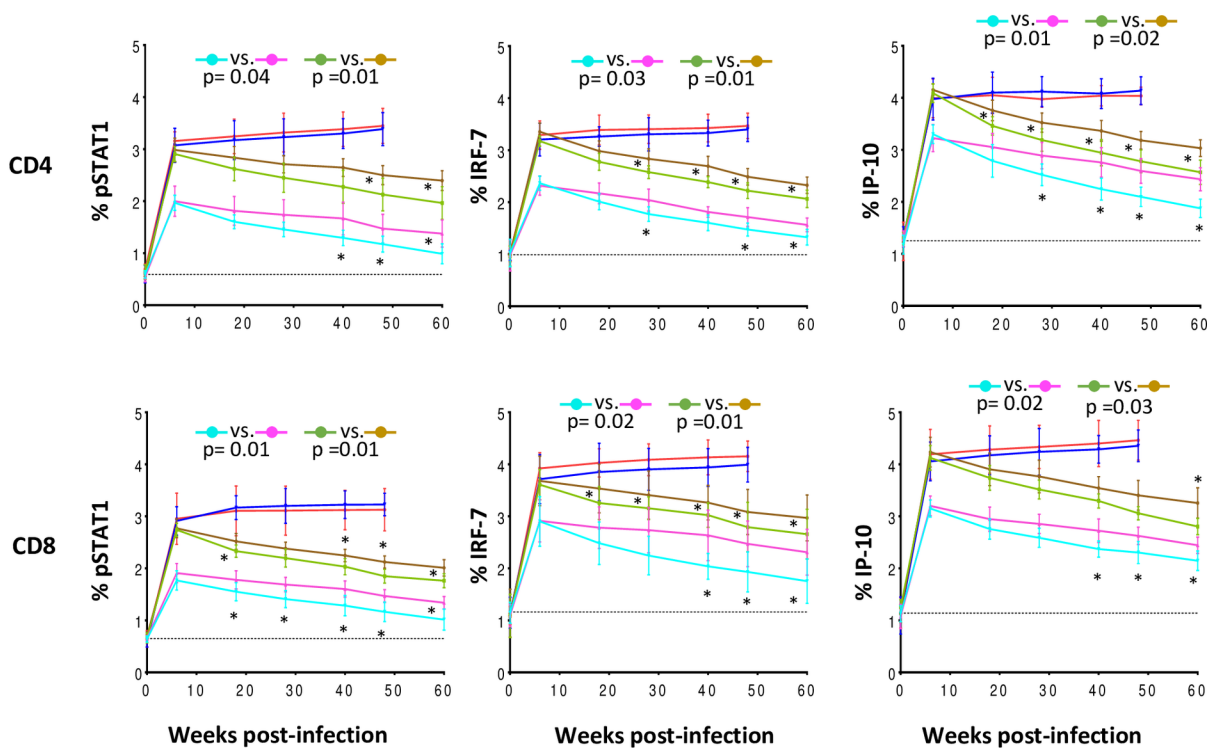

Supplement: S1 Fig — A. Gating strategy for ICS to evaluate percentages of IRF-7, pSTAT1 and IP-10 in PBMC. Columns report analyses for: 1. Singlets; staining for: 2. Live/Dead, 3. IRF7, 4. pSTAT1, 5. IP-10. First row shows fluorescence minus one staining (FMO) in one representative sample for IRF7, pSTAT1, IP-10. Absence of the antibody indicated above was used to position gates to evaluate percentage of positive cells for that specific antibody. Rows 2–5 show staining for IRF7, pSTAT1, IP-10 in one representative sample for each group. B. Table report average group percentages of PBMC subpopulations on week 6 when inhibitor therapy was initiated and on week 60 when it was stopped. Differences were minimal between paired groups (Group 3 and 4 and Groups 5 and 6). C. Percentages of CD4+ and CD8+ T cells in PBMC expressing pSTAT1, IRF7, and IP-10. D. Percentages of CD4+ and CD8+ T cells in lymph node MNC expressing pSTAT1, IRF7, and IP-10. The black, dotted line indicates the average of the 32 animal values measured in samples obtained on the day before infection. The reported p values were calculated for the comparison of the AUC from the first time point available after p38 MAPK inhibitor treatment initiation to 60 and refer to AUC comparisons in paired groups. Between group comparisons at individual time points were carried out with Wilcoxon-Mann-Whitney (rank sum) test. Asterisks mark significant time point comparisons for Group 3 vs. Group 4 (asterisks above brown line) or Group 5 vs. Group 6 (asterisks below blue line). (PDF) [file ppat.1007268.s001.pdf]

**Supplemental Figure 2**

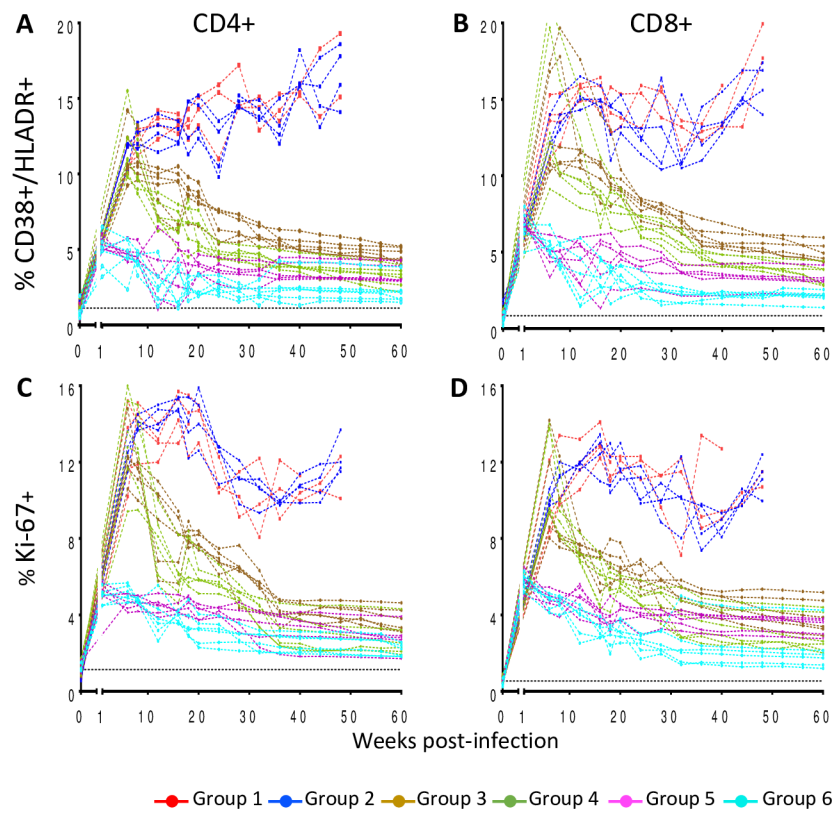

Supplement: S2 Fig — Percentages of HLA-DR+/CD38+ in CD4+ (A) and CD8+ (B) T cells and of Ki-67+ in CD4+ (C) and CD8+ (D) T cells in PBMC. Data are reported for each individual animal. The black, dotted line indicates the average of all 32 individual animal values measured before infection. The reported p values were calculated for comparisons of AUC between week 8 and 60 in paired groups. (PDF) [file ppat.1007268.s002.pdf]

### Supplemental Figure 3

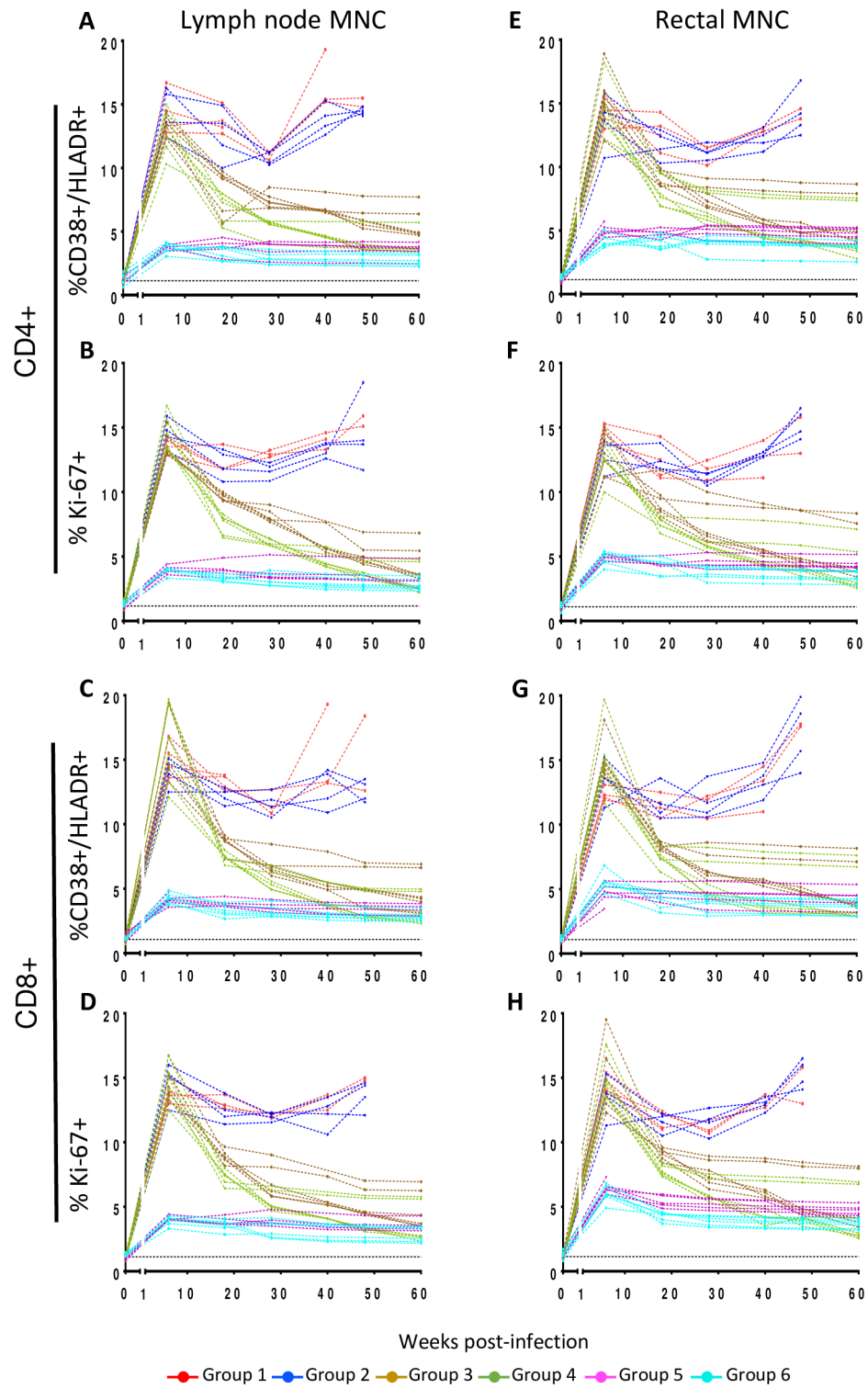

Supplement: S3 Fig — Data for lymph node and rectal tissue T-cell expression of immune activation markers in biopsies collected at each PH-797804 treatment cycle start and end time points are shown. Panels report percentages HLA-DR+/CD38+/CD4+ (A) or Ki-67+/CD4+ T cells (B) in inguinal lymph nodes and in rectal mucosa (E and F, respectively), percentage of HLA-DR+/CD38+/CD8+ (C) or Ki-67+/CD8+ T cells (D) in lymph nodes and in rectal mucosa (G and H, respectively). Data are represented for each individual animal. The black, dotted line indicates the average of all 32 individual animal values measured before infection. The reported p values were calculated for comparisons of AUC between week 18 (first available time point after beginning of PH-797804 treatment) to 60. (PDF) [file ppat.1007268.s003.pdf]

Supplemental Figure 4

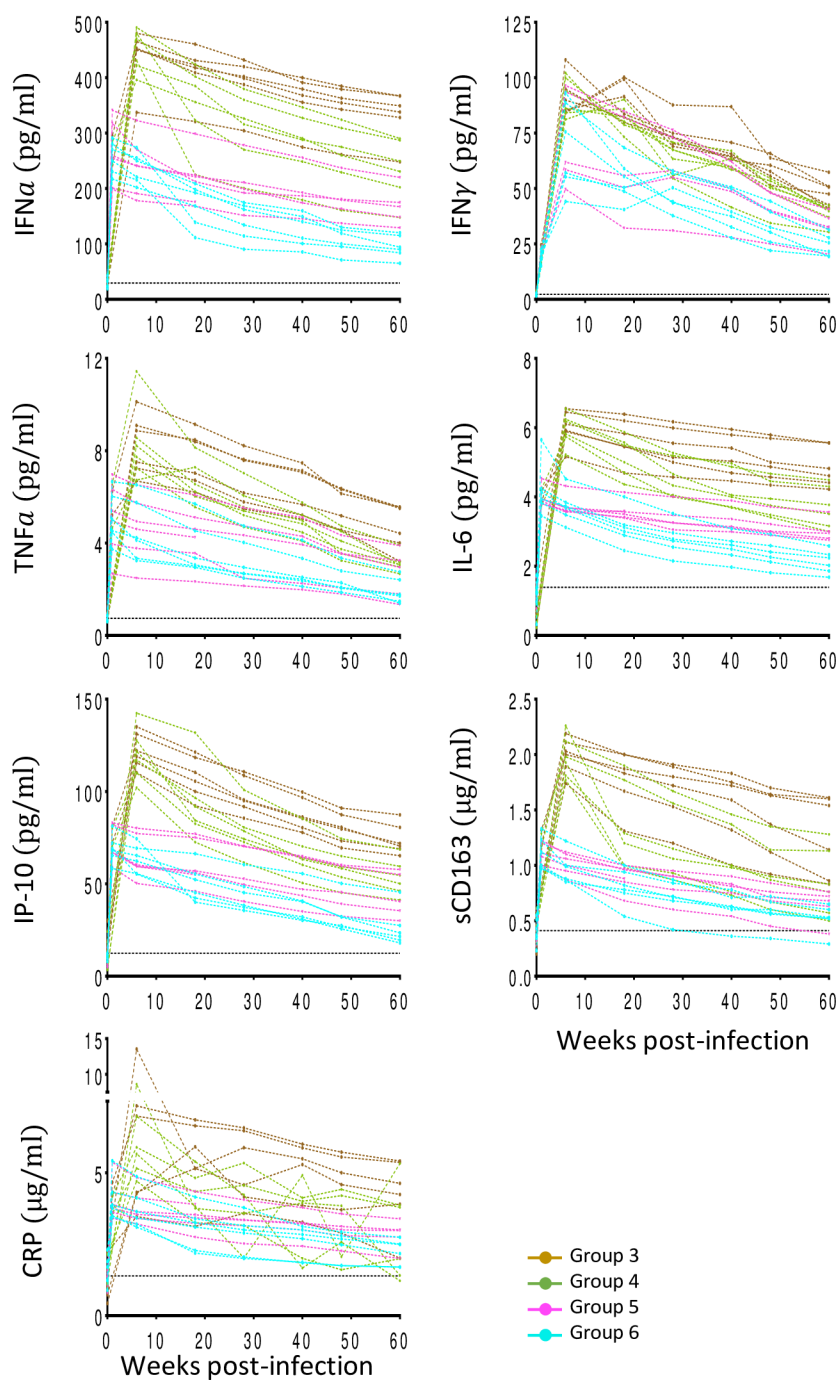

Supplement: S4 Fig — Longitudinal assessment of inflammatory cytokines levels in plasma of IFNα, IFNγ, TNFα, IL-6, IP-10 (pg/ml) and inflammatory markers CRP and sCD163 (μg/ml) by ELISA. Data are represented for each individual animal. The reported p values were calculated for comparisons of AUC between week 18 and 60 in paired groups. (PDF) [file ppat.1007268.s004.pdf]

**Supplemental Figure 5**

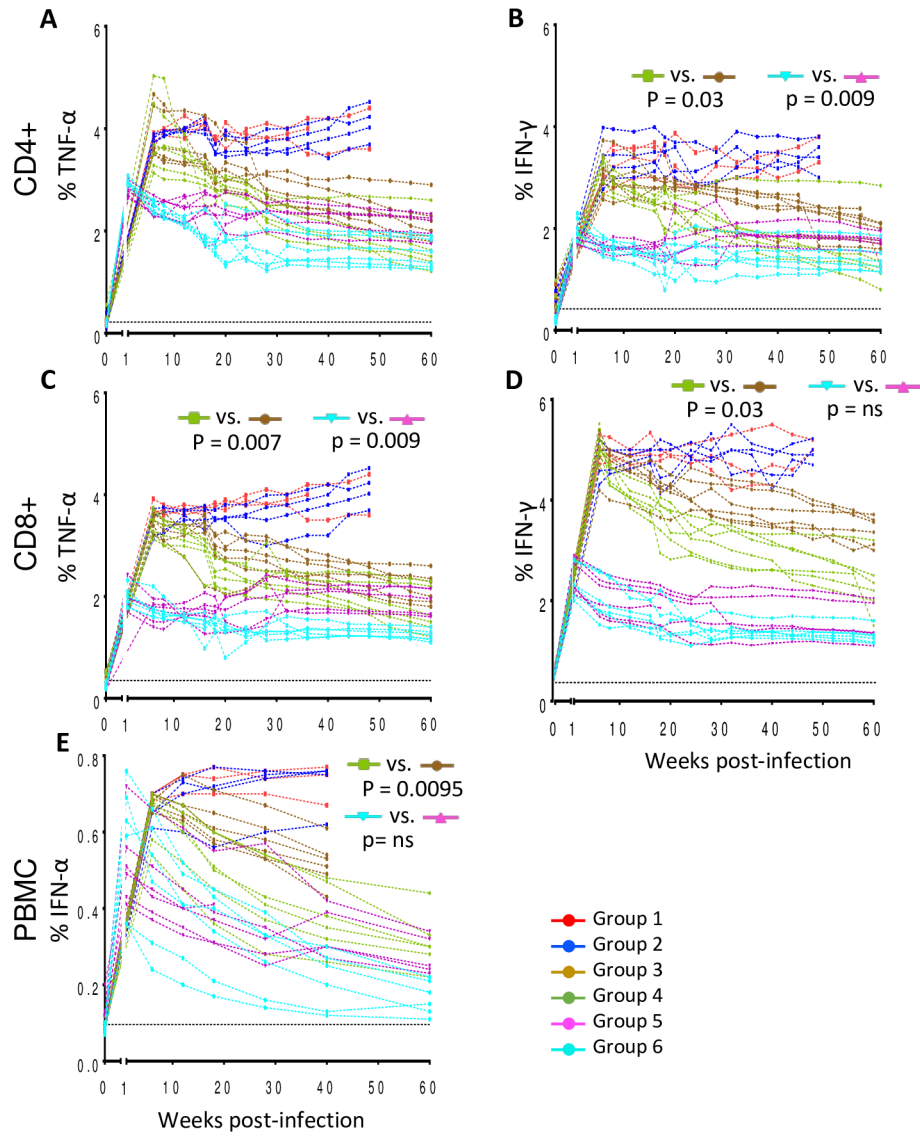

Supplement: S5 Fig — Longitudinal analysis of frequency of CD4+ T cells expressing TNFα (A) and IFNγ (B) and of CD8+ T cells expressing TNFα (C), IFNγ (D), as detected in unstimulated, fresh PBMC obtained from animals after bleeding. E. Percentages of INFα+ cells in total PBMC. Data are reported for each individual animal. The black, dotted line indicates the average of all 32 individual animal values measured before infection. The reported p values were calculated for comparisons of AUC between week 8 and 60 in paired groups. (PDF) [file ppat.1007268.s005.pdf]
